# Acute dose-dependent effects of 4-bromo-2,5-dimethoxyphenethylamine (2C-B) compared with 3,4-methylenedioxymethamphetamine (MDMA) and psilocybin in a double-blind, placebo-controlled study in healthy participants

**DOI:** 10.1038/s41386-026-02428-9

**Published:** 2026-04-28

**Authors:** Denis Arikci, Joran Borgulya, Isabelle Straumann, Patrick Vizeli, Dino Luethi, Jan Thomann, Deborah Rudin, Ina Vukalovic, Anne Eckert, Matthias E. Liechti, Friederike Holze

**Affiliations:** 1 Clinical Pharmacology and Toxicology, Department of Biomedicine and Department of Clinical Research, University Hospital Basel, Basel, Switzerland; 2 Department of Pharmaceutical Sciences, University of Basel, Basel, Switzerland; 3 Psychiatric University Hospital, University of Basel, Basel, Switzerland; 4 Transfaculty Research Platform Molecular and Cognitive Neuroscience, University of Basel, Basel, Switzerland

**Keywords:** Drug development, Predictive markers

## Abstract

Based on its in vitro profile and preliminary evidence, 4-bromo-2,5-dimethoxyphenethylamine (2C-B) may have psychoactive properties that are similar to 3,4-methylenedioxymethamphetamine (MDMA) and psilocybin, which are investigated for the treatment of posttraumatic stress disorder and depressive disorders. We compared acute effects of 2C-B (10, 20, and 30 mg), 125 mg MDMA, and 25 mg psilocybin in 24 healthy participants (12 women, 12 men) using a double-blind, randomized, placebo-controlled, crossover design. Outcome measures included acute subjective effects, autonomic effects, adverse effects, effects on emotional and cognitive empathy, plasma oxytocin and neurophysin I concentrations, and pharmacokinetics up to 9 h. 2C-B produced dose-dependent subjective effects, with the 30 mg dose exerting comparable “any drug effects” to MDMA but lower “any drug effects” than psilocybin. Only psilocybin induced “bad drug effects” and “anxiety” compared with placebo. The 30 mg dose of 2C-B induced psychedelic-type alterations of state of consciousness and increased emotional empathy similarly to MDMA. The average subjective effect duration of 30 mg 2C-B was 4.9 h and similar to MDMA (4.8 h) and shorter than psilocybin (6.1 h). MDMA produced the highest cardiovascular stimulation, followed by psilocybin and 2C-B. Only MDMA increased plasma oxytocin and neurophysin I concentrations. 2C-B exhibited dose-proportional pharmacokinetics, with a plasma elimination half-life of ~1.3 h. The 30 mg dose of 2C-B induced entactogenic and psychedelic effects similarly to MDMA and psilocybin, respectively. MDMA is more cardiostimulant than psilocybin and 2C-B. At the tested dose-level, psilocybin is more distressing than MDMA and 2C-B. These results may assist with dose-finding for future 2C-B research and provide a direct comparison with standard doses of the prototypical compounds MDMA and psilocybin. Trial registration: ClinicalTrials.gov identifier: NCT05523401.

## Introduction

Psychedelics, including psilocybin and 3,4-methylenedioxymethamphetamine (MDMA) are currently under investigation for the treatment of several psychiatric disorders [1–6]. 4-Bromo-2,5-dimethoxyphenethylamine (2C-B) is a phenethylamine that was first synthesized by A. Shulgin in 1974 [7] and is widely used recreationally [8]. 2C-B acts mainly as a 5-hydroxytryptamine-2A (5-HT_2A_) receptor agonist [9] similarly to the classic psychedelic psilocybin, which induces its acute effects in humans primarily via 5-HT_2A_ receptor stimulation [10]. 2C-B also interacts with serotonin transporters in vitro similar to the prototypical entactogen 3,4-methylenedioxymethamphetamine (MDMA), although only with low potency [9]. MDMA and psilocybin are both currently investigated for the treatment of posttraumatic stress disorder and depressive disorders [1–6], and 2C-B may also have therapeutic potential [11].

Acute effects of 2C-B have been anecdotally described to be similar to MDMA when used at low to moderate doses (5–10 mg 2C-B) and more similar to serotonergic psychedelics when used at high doses [12–16]. Medium and high doses of 2C-B were defined as 10–25 mg and 25–40 mg, respectively [15, 16]. In recreational 2C-B users who self-administered 20 mg 2C-B and completed subjective effect measurements and neuropsychological testing, the acute effect profile was reported to be entactogenic with psychedelic/hallucinogenic properties based on a pattern of findings including reduced anger on the Profile of Mood States (POMS), increased well-being and psychedelic-type effects on Visual Analog Scales (VAS) and impaired recognition of happy faces on the facial emotion recognition task (FERT) [17]. In a small observational study, experienced drug users self-administered a dose of 10, 15, or 20 mg 2C-B, and acute effects were described as psychedelic/psychostimulant-like, with mood changes that were reported to be more prominent than perceptual changes indicated by findings including VAS ratings for feeling high, good effects, liking, and perceptual changes; elevated euphoria and amphetamine-like effects on the Addiction Research Center Inventory (ARCI); and ratings of intensity, affect, and altered volition on the Hallucinogen Rating Scale (HRS) [16]. A double-blind crossover study in healthy psychedelic-experienced participants showed largely comparable, although weaker and shorter, effects of a 20 mg dose of 2C-B on the 5 Dimensions of Altered States of Consciousness (5D-ASC) compared with 15 mg psilocybin and no empathogenic effects on the Multifaceted Empathy Test (MET) [18]. Altogether, the findings suggest that 2C-B has mixed entactogenic and psychedelic-type properties. However, previous controlled studies in humans have only compared its effects with psilocybin and not with MDMA and have only investigated 2C-B doses up to 20 mg [18]. Therefore, the present study aimed to characterize the acute subjective, empathogenic, autonomic, and endocrine responses to 10, 20, and 30 mg 2C-B in healthy participants and in direct comparison with prototypical substances that show overlapping receptor profiles, such as the entactogen MDMA (125 mg) and the psychedelic psilocybin (25 mg) at representative doses to investigate how their in vitro profiles translate into pharmacodynamic effects in healthy participants using well-established and sensitive tools.

## Methods and materials

### Study design

The present study used a double-blind, placebo-controlled, random-order, crossover design with six experimental test sessions to investigate acute responses to (i) placebo, (ii) 10 mg 2C-B, (iii) 20 mg 2C-B, (iv) 30 mg 2C-B, (v) 125 mg MDMA, and (vi) 25 mg psilocybin. Washout periods between test days were at least 10 days. The study was conducted in accordance with the Declaration of Helsinki and International Conference on Harmonization Guidelines in Good Clinical Practice and approved by the Ethics Committee of Northwest Switzerland (EKNZ) and the Swiss Federal Office for Public Health. The study was registered at ClinicalTrials.gov (NCT05523401).

### Participants

Twenty-four healthy participants (12 men and 12 women; mean age ± SD: 36 ± 9 years; range: 25–52 years; mean body weight ± SD: 68 ± 12 kg; range: 51–91 kg) were recruited from volunteers who had contacted our research group with interest in participating in a psychedelic trial via advertisement on the website of the University of Basel. All participants provided written informed consent and were paid for their participation. Detailed information about the exclusion and inclusion criteria are outlined in the Supplemental Information. Sample characteristics and substance use history of the participants are summarized in Supplementary Table S1.

### Study drugs

Detailed information about the study drugs and the exact content can be found in the Supplemental Information. At the end of each session and at the end of the study, the participants were asked to retrospectively guess their treatment assignment by indicating the exact treatment condition.

### Study procedures

Detailed information about the study procedures can be found in the Supplemental Information. The study included a screening visit, six 10-h test sessions, and an end-of-study visit. The screening visit included the informed consent process, where participants were informed about the study design and potential effects and risks of MDMA, 2C-B, and psilocybin. Test sessions began at 8:00 AM, outcome measures were repeatedly assessed for 9 h, and the participants were released ~9 h after drug administration.

### Subjective drug effects

Subjective effects were assessed repeatedly using Visual Analog Scales (VASs) [19–22] before and 0, 0.5, 1, 1.5, 2, 2.5, 3, 3.5, 4, 5, 6, 7, 8, and 9 h after drug administration. The Adjective Mood Rating Scale (AMRS) [23] was administered before and 3, 6, and 9 h after drug administration. The 5 Dimensions of Altered States of Consciousness (5D-ASC) scale [24–26] and Psychedelic Experience Scale (PES), which includes the Mystical Effect Questionnaire (MEQ) [27–29], were administered 9 h after drug administration to retrospectively rate peak psychedelic and mystical-type effects. A detailed description of the psychometric assessments can be found in the Supplementary Information.

Time to onset, maximal effect, time to maximal effect, time to offset, effect duration, and area under the effect-time curve were assessed using individual effect-time plots of the VAS item “any drug effect,” with a threshold of 10% of the maximum response. For maximal ratings <50% of the threshold was set to 5. The analyses were conducted using Phoenix WinNonlin 8.7 (Certara, Princeton, NJ, USA).

### Autonomic and adverse effects

Blood pressure, heart rate, and tympanic body temperature were repeatedly measured before and 0, 0.5, 1, 1.5, 2, 2.5, 3, 3.5, 4, 5, 6, 7, 8, and 9 h after drug administration [30]. Adverse effects were assessed before and 9 h after drug administration using the List of Complaints [31]. Additionally, an electrocardiogram (ECG) was obtained before and 2 h after drug administration.

### Plasma concentrations and pharmacokinetic analyses

Blood was collected in lithium heparin tubes before and 0.5, 1, 1.5, 2, 2.5, 3, 3.5, 4, 5, 6, 7, 8, and 9 h after drug administration. Samples were centrifuged immediately, and plasma was stored at –80 °C until analysis. Plasma concentrations were determined by fully validated high-performance liquid chromatography-tandem mass spectrometry [32]. All pharmacokinetic analyses were conducted using Phoenix WinNonlin 8.7 (Certara, Princeton, NJ, USA). Pharmacokinetic parameters were estimated using non-compartmental methods.

### Assessment of social cognition

*Multifaceted Empathy Test (MET)*: The MET is a reliable and valid task that is used to assess cognitive and emotional aspects of empathy [33] and has previously been shown to be sensitive to MDMA [34, 35], LSD [36, 37], and psilocybin [38]. A detailed description can be found in the Supplementary Information. The MET was performed 3 h after drug administration.

*Facial Emotion Recognition Task (FERT)*: The FERT is a widely used task to assess the ability to recognize emotions from facial expressions. The task has previously been shown to be sensitive to psychedelics [37], MDMA [34, 35, 39, 40], and stimulants [35, 40, 41]. A detailed description can be found in the Supplementary Information. The FERT was performed after the MET ~ 3 h after drug administration.

### Endocrine effects

Plasma oxytocin and neurophysin I samples were collected in ethylenediaminetetraacetic acid tubes before and 1.5, 3, and 6 h after drug administration. Oxytocin was measured as previously described [36]. Neurophysin I was measured using the in vitro Oxytocin-Neurophysin I Prepropeptide SimpleStep ELISA kit (Abcam, Cambridge, UK) according to the manufacturer’s protocol [42].

### Data analysis

Peak maximum effect (E_max_) and/or minimum effect (E_min_) or peak change from baseline (ΔE_max/min_) values were determined for repeated measures over time. Within-subject differences across conditions were analyzed using repeated-measures ANOVA (rmANOVA). Pairwise *post hoc* comparisons were performed with Tukey correction for multiple comparisons. Effect sizes for condition effects are reported as partial eta squared (η_p_²), and effect sizes for pairwise comparisons as Cohen’s d_z_, calculated as the mean paired difference divided by the standard deviation of the paired differences. All statistical analyses were conducted using R 2025.09.2 software (RStudio, PBC, Boston, MA, USA). The criterion for significance was *p* < 0.05.

## Results

### Subjective drug effects

**Fig. 1:**
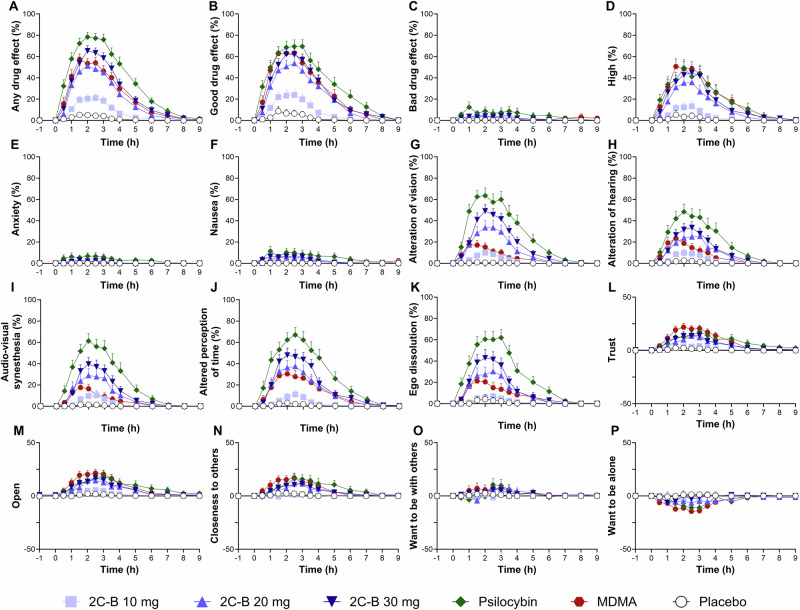
Acute subjective effects over time on the Visual Analoge Scale (VAS). **A**–**P** Acute subjective effects of 2C-B, MDMA, and psilocybin over time. Drug or placebo was administered at t = 0 h. 2C-B dose-dependently increased effects on all VASs except for (**D**) “bad drug effect” and **E** “anxiety,” with the highest dose of 2C-B (30 mg) inducing similar (**A**) “any drug effects” as MDMA but weaker effects than psilocybin. The highest dose of 2C-B, MDMA, and psilocybin induced similar (**B**) “good drug effects,” and only psilocybin induced ratings of (**D**) “bad drug effects” and **E** “anxiety” compared with placebo. The highest dose of 2C-B induced psychedelic-like effects on the VASs (**G**) “alteration of vision,” (**H**) “alteration of hearing,” (**I**) “audio-visual synesthesia,” (**J**) “altered perception of time,” and (K) “ego dissolution,” which were weaker than psilocybin but stronger than dose induced by MDMA. The 10 mg dose of 2C-B showed no significant increases nor decreases in any of the bidirectional VAS items compared with placebo. The 20 and 30 mg doses of 2C-B, MDMA, and psilocybin increased the items (**L**) “trust”, (**M**) “open”, (**N**) “closeness to others” and (**O**) “Want to be with others” compared with placebo. The 20 and 30 mg doses of 2C-B, and psilocybin also induced significant increases in (**P**) “Want to be alone” compared with placebo. The data are expressed as the mean ± SEM (A-K) percentage of maximally possible scale scores and (**L**–**P**) scores on a bidirectional scale from -50 – +50 in 24 participants. The corresponding maximal responses and statistics are shown in Supplementary Table S2.

**Fig. 2:**
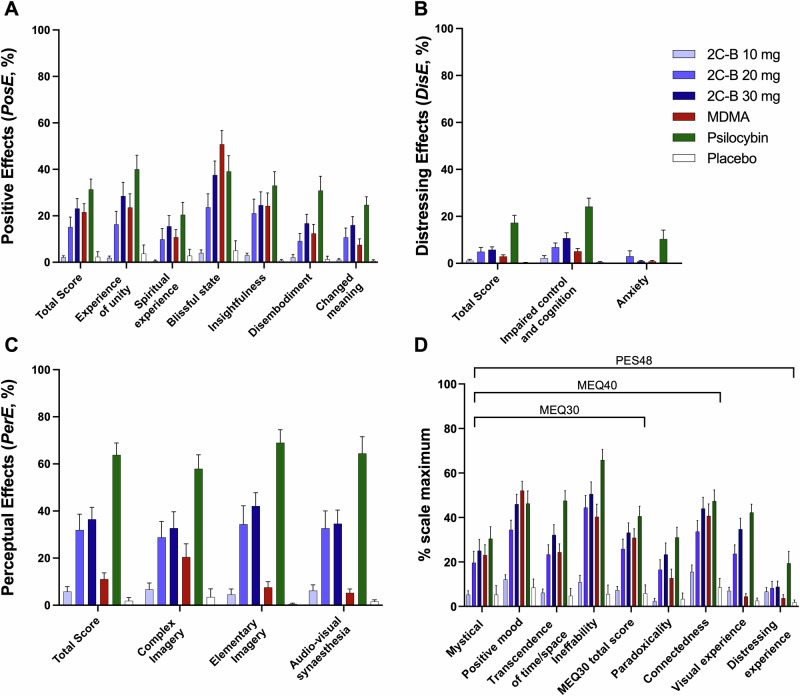
Acute alterations of mind and mystical-type experiences. **A**–**C** Acute alterations of mind on the Altered States of Consciousness (3D-ASC) and **D** mystical-type experiences on the Mystical Effect Questionnaire (MEQ) and Psychedelic Experience Scale (PES). 2C-B dose-dependently induced alterations of state of consciousness, with the highest dose of 2C-B inducing positive (**A**) and distressing (**B**) effects that were similar to MDMA while inducing significantly higher perceptual effects (**C**) than MDMA. Compared with psilocybin, 2C-B induced lower psychedelic-type ratings, such as disembodiment, distressing effects, and perceptual effects. **D** 2C-B dose-dependently induced mystical-type experiences, with the highest dose inducing similar effects as MDMA and psilocybin, reflected by the MEQ30 total score. Psilocybin induced higher ratings of transcendence of time/space and ineffability compared with the highest dose of 2C-B and MDMA. On the PES, the highest dose of 2C-B and psilocybin induced significantly higher ratings of “visual experience” compared with MDMA. The data are expressed as the mean ± SEM percentage of maximally possible scale scores in 24 participants. The corresponding maximal responses and statistics are shown in Supplementary Table S5 and S5.

Subjective effects over time on the VAS and AMRS are shown in Fig. 1 and Supplementary Fig. S1, respectively. Characteristics of the acute response, including time to onset, time to offset, and effect duration, are presented in Table 1. The corresponding peak responses and statistics are presented in Supplementary Table S2-S3. Alterations of mind and mystical-type effects are shown in Fig. 2. Statistics are summarized in Supplementary Table S4-S5.

**Table 1:** Characteristics of the acute subjective response (*n* = 24).

Effect	2C-B 10 mg	2C-B 20 mg	2C-B 30 mg	MDMA 125 mg	Psilocybin 25 mg
Time to onset (h)	0.8 ± 0.5^a^ (0.3–2.3)	0.6 ± 0.3 (0.1–1.3)	0.5 ± 0.3 (0.1–1.2)	0.5 ± 0.3 (0.1–1.2)	0.3 ± 0.2 (0.1–0.8)
Time to offset (h)	4.0 ± 1.4^a^ (1.0–6.9)	5.2 ± 1.2 (3.2–7.5)	5.4 ± 1.5 (1.9–8.5)	5.3 ± 1.4 (2.6–8.0)	6.4 ± 1.3 (3.7–8.9)
Time to maximal effect (h)	2.1 ± 0.8^a^ (0.5–3.0)	2.0 ± 0.6 (1.0–3.5)	2.3 ± 0.7 (1.0–3.5)	1.9 ± 0.9 (0.5–4.0)	2.1 ± 0.7 (1.0–3.3)
Effect duration (h)	3.1 ± 1.5^a^ (0.7–6.3)	4.6 ± 1.4 (2.1–6.9)	4.9 ± 1.5 (1.2–8.0)	4.8 ± 1.3 (2.1–7.3)	6.1 ± 1.3 (3.4–8.5)
Maximal effect (%)	27 ± 20 (0–66)	56 ± 26 (15–100)	72 ± 22 (14–100)	70 ± 21 (22–100)	87 ± 18 (41–100)
AUEC (% * h)	60 ± 58 (0–197)	168 ± 112 (23–462)	226 ± 106 (21–511)	203 ± 98 (46–414)	238 ± 125 (108–564)

Parameters are for “any drug effect” as determined using the individual effect-time curves. The threshold to determine times to onset and offset was set to 10% of the individual maximal response. Values are mean ± SD (range); AUEC, area under the effect curve.

^a^*n *= 20.

On the VAS, 2C-B produced dose-dependent subjective responses starting at the 10 mg dose, which already produced significant but low “any drug effects” compared with placebo (*p* < 0.01). The 30 mg dose produced “any drug effects” that were comparable to 125 mg MDMA but significantly weaker than 25 mg psilocybin (*p* < 0.05). The 30 mg dose of 2C-B, 125 mg MDMA, and 25 mg psilocybin produced similar “good drug effects” and “high,” and only psilocybin produced weak but significant increases in “bad drug effects” and “anxiety” compared with placebo (both *p* < 0.001). Psychedelic-like effects on the VASs “alteration of vision”, “alteration of hearing”, “audio-visual synesthesia,” “altered perception of time,” and “ego dissolution” were significantly increased by the 20 and 30 mg doses of 2C-B, MDMA, and psilocybin compared with placebo (all *p* < 0.001 except for “alteration of vision” and “audio-visual synesthesia” for MDMA, both *p* < 0.01). Psilocybin also produced significantly higher effects compared with MDMA (all *p* < 0.001) and higher ratings than 30 mg 2C-B on all subscales except for “altered perception of time.” The 30 mg dose of 2C-B produced significantly higher ratings on the VASs “alteration of vision,” “audio-visual synesthesia,” and “ego dissolution” compared with MDMA (*p* < 0.001, *p* < 0.05, and *p* < 0.05, respectively). On the bidirectional VASs that measure interpersonal relations, the 10 mg dose of 2C-B showed no significant increases nor decreases compared with placebo (all *p* > 0.05). The 20 and 30 mg doses of 2C-B, MDMA, and psilocybin increased the items “trust” (all *p* < 0.001), “open” (all *p* < 0.001), “closeness to others” (*p* < 0.01 for 20 mg of 2C-B and *p* < 0.001 for all others), and “want to be with others” (*p* < 0.05 for 20 and 30 mg of 2C-B and *p* < 0.001 for MDMA and psilocybin) compared with placebo. The 30 mg dose of 2C-B (*p* < 0.01), and psilocybin (*p* < 0.001) also induced significant increases in “want to be alone” compared with placebo.

2C-B dose-dependently induced alterations of state of consciousness on the 5D-ASC, with 30 mg 2C-B inducing similar alterations of mind compared with MDMA but weaker alterations of mind than psilocybin based on the 5D-ASC and 3D-ASC total scores (both *p* < 0.001). MDMA induced only positively valenced effects, including higher ratings on the subscales “experience of unity” (*p* < 0.01), “blissful state” (*p* < 0.001), and “insightfulness” (*p* < 0.001), compared with placebo, whereas 30 mg 2C-B and psilocybin induced more complex and psychedelic-like alterations of state of consciousness by also inducing perceptual effects (both *p* < 0.001) and psilocybin also inducing distressing effects (*p* < 0.001). Only psilocybin increased ratings on the subscales “spiritual experience” (*p* < 0.01) and “anxiety” (*p* < 0.01) compared with placebo. Psilocybin overall produced significantly higher distressing effects and perceptual changes compared with 30 mg 2C-B and MDMA (both *p* < 0.001). MDMA, 30 mg 2C-B, and psilocybin produced statistically similar positive effects. Findings on the MEQ30 and PES showed a similar pattern to the 5D-ASC (Fig. 2D).

### Autonomic and adverse effects

**Fig. 3:**
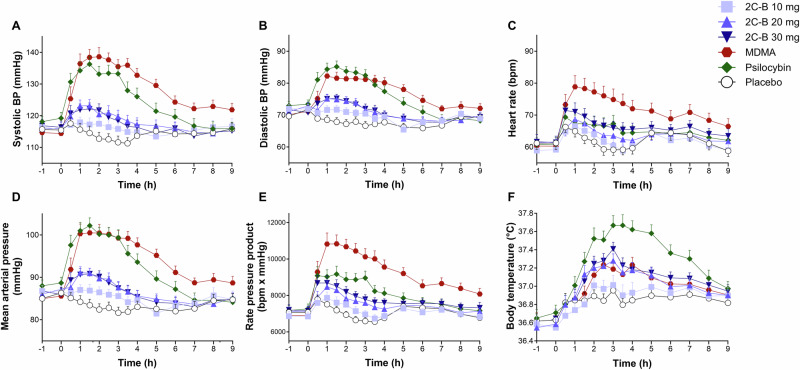
Acute autonomic effects. **A**–**F** Acute autonomic effects over time. Doses of 10, 20, and 30 mg 2C-B increased (**A)** systolic and **B** diastolic blood pressure dose-dependently but only marginally compared with placebo, whereas MDMA and psilocybin markedly increased blood pressure compared with placebo and all 2C-B doses. 2C-B showed an increase in (**C**) heart rate only at the highest dose similarly to psilocybin and lower than MDMA. Overall, the highest dose of 2C-B and psilocybin produced similar acute cardiovascular stimulation, reflected by the (**E**) rate pressure product, that was significantly lower than MDMA. **F** Psilocybin produced a significantly larger increase in body temperature compared with MDMA and the highest dose of 2C-B. All increases in autonomic effects were transient. Drug or placebo was administered at t = 0 h. The data are expressed as the mean ± SEM in 24 subjects. Maximal effects and statistics are shown in Supplementary Table S2.

Autonomic effects over time and respective peak effects are shown in Fig. 3 and Supplementary Table S2, respectively. Frequently reported adverse effects are presented in Supplementary Table S6. 2C-B dose-dependently but only mildly increased blood pressure, with 30 mg 2C-B inducing significantly lower increases compared with MDMA and psilocybin (*p* < 0.001). Psilocybin and MDMA induced comparable increases in blood pressure. Only 30 mg 2C-B and not the lower doses increased heart rate compared with placebo (*p* < 0.01), and these increases were similar to psilocybin but lower than MDMA (*p* < 0.001). Overall, MDMA induced the highest cardiovascular stimulation, followed by psilocybin and 2C-B, reflected by the rate pressure products. Psilocybin (*p* < 0.001) and 20 and 30 mg 2C-B (both *p* < 0.05) but not MDMA increased body temperature compared with placebo. None of the drugs had an effect on QT-time. All conditions, except 10 mg 2C-B, increased the total acute (0-9 h) adverse effect score on the List of Complaints compared with placebo (all *p* < 0.001). No severe adverse events were observed.

A total of two participants dropped out of the study after inclusion. One participant withdrew from study participation after a challenging psilocybin experience in the first study session. The other participant withdrew consent before the first study session.

### Plasma concentrations

Pharmacokinetic parameters are shown in Table 2. Concentration-time curves are shown in Supplementary Fig. S2–S4.

**Table 2:** Pharmacokinetic parameters after oral administration based on non-compartmental analyses [geometric mean (95% CI), range], *N* = 24.

Drug and Dose (mg)	Parameter	C_max_ (ng/ml)	t_max_ (h)	t_1/2_ (h)	AUC_9_ (ng·h/ml)	AUC_∞_ (pg·h/ml)
10 mg 2C-B	2C-B	2.5 (2.2–2.8)	1.8 (1.6–2.0)	1.3 (1.1–1.6)	6.2 (5.3–7.1)	7.7 (6.9–8.6)
		1.1–4.6	1.0–3.0	0.81–2.7	1.8–8.8	4.4–11
20 mg 2C-B	2C-B	4.6 (4.1–5.1)	1.9 (1.7–2.2)	1.3 (1.2–1.5)	14 (12–15)	15 (14–17)
		3.2–7.8	1.0–3.5	0.88–2.2	7.8–23	9.5–24
30 mg 2C-B	2C-B	6.4 (5.8–7.0)	2.1 (1.8–2.5)	1.3 (1.2–1.4)	21 (19–24)	23 (21–25)
		3.5–9.2	1.5–4.0	0.9–2.1	13–35	15–36
25 mg Psilocybin	Psilocin	19 (17–21)	2.5 (2.2–2.8)	2.0 (1.9–2.2)	84 (75–93)	92 (83–102)
		11–30	1.5–4.0	1.5–2.9	53–131	59–146
125 mg MDMA	MDMA	260 (239–283)	3.0 (2.6–3.6)	9.4 (8.4–11)	1690 (1557–1834)	4078 (3609–4608)
		175–348	1.5–5.0	5.5–15	1123–2204	2616–3825

### Social cognition

Effects on empathy on the MET are presented in Supplementary Fig. S5. Effects on emotion recognition on the FERT are presented in Fig. S6. On the MET, MDMA and 30 mg 2C-B both increased explicit emotional empathy in response to positive images (*p* < 0.05) compared with placebo. Only psilocybin decreased cognitive empathy in response to positive (*p* < 0.001) and negative (*p* < 0.1) stimuli compared with placebo. There were no significant effects on implicit emotional empathy and to negative stimuli by any of the drugs. On the FERT, psilocybin reduced the overall correct identification of emotions compared with placebo (*p* < 0.05) and significantly reduced the response to fearful stimuli compared with placebo (*p* < 0.01). 2C-B and MDMA did not significantly change emotion recognition.

### Oxytocin and neurophysin I levels

Effects on plasma oxytocin and neurophysin I levels are presented in Fig. S7–S8. Only MDMA increased oxytocin and neurophysin I levels compared with placebo, 2C-B, and psilocybin (*p* < 0.001).

### Blinding

Data on the participants’ retrospective identification of the drug conditions are shown in Supplementary Table S7. The participants could not correctly identify 2C-B or psilocybin immediately after the study session ( ~ 50% correct), whereas MDMA and placebo were correctly identified by 79% and 67% of the participants, respectively. After the study, identification accuracy increased overall. However, only 38% of the participants identified the high dose of 2C-B correctly, and only 52% identified psilocybin correctly, whereas MDMA and placebo were correctly identified by 79%. Assessment of drug identification 2 h after drug administration showed only limited accuracy.

## Discussion

The present study investigated acute effects of three different doses of 2C-B in healthy participants compared with the psychedelic psilocybin and entactogen MDMA. Previous studies only used one single dose level per participant and used lower doses of 2C-B, and they only compared effects of 2C-B with psilocybin and not with MDMA [16–18]. We used three doses of 2C-B within the same participant that extended the range of previously investigated doses. We covered the range of reportedly therapeutically meaningful doses, with 10 mg 2C-B considered a low dose, 20 mg considered a medium dose that induces typical psychedelic and entactogenic effects, and 30 mg considered a high dose that is thought to induce strong effects, sometimes even negative effects [15, 16]. The present study was also the first to fully characterize the pharmacokinetics of 2C-B, including different dose levels and a validated analytical method [32]. Lastly, we present the first within-subjects comparison of psilocybin and MDMA at therapeutically relevant doses.

2C-B induced dose-dependent subjective effects, with the 10 mg dose producing only very mild effects and almost no psychedelic-type alterations of state of consciousness, the 20 mg dose producing mild but significant alterations of state of consciousness, and 30 mg producing an overall drug intensity (“any drug effect”) that was similar to 125 mg MDMA but lower than 25 mg psilocybin. Ratings of “good drug effect” or “high,” however, were comparable between the highest dose of 2C-B, psilocybin, and MDMA. The highest dose of 2C-B also induced less psychedelic-specific effects on the VAS, including “alteration of vision,” “alteration of hearing,” “audio-visual synesthesia,” “altered perception of time,” and “ego dissolution,” compared with psilocybin, but stronger effects than MDMA. Effects on questionnaires that specifically characterize psychedelic alterations of state of consciousness, such as the 5D-ASC and MEQ30, mirrored these effects. The 30 mg dose of 2C-B induced numerically lower but statistically nonsignificantly different positive effects on the 5D-ASC compared with psilocybin. These findings indicate that a relatively high dose of 30 mg 2C-B induces strong positive alterations of state of consciousness that are similar to the prototypical serotonergic psychedelic psilocybin but with lower ratings of perceptual effects and distressing effects compared with a standard dose of 25 mg psilocybin. In a previous crossover study that compared 15 mg psilocybin with 20 mg 2C-B, both drugs showed similar peak drug intensities across different VASs, but 15 mg psilocybin produced overall stronger effects on the 5D-ASC compared with 2C-B [18]. Altogether, the present and previous findings indicate that 2C-B induces psychedelic effects that are clearly dose-dependent up to a dose of 30 mg. We did not test higher doses of 2C-B, but speculate, based on the herein reported dose-response relationship of 2C-B, that 40 mg 2C-B might have induced similar alterations of consciousness, including distressing effects, compared with 25 mg psilocybin.

Furthermore, we measured effects of 2C-B on social cognition using well established tasks, such as the MET and FERT. On the MET, 30 mg 2C-B and MDMA increased explicit emotional empathy in response to positive stimuli compared with placebo, indicating similar empathogenic effects of 2C-B and MDMA [34, 35]. In contrast, a previous study found no effect of a 20 mg dose of 2C-B on the MET [18]. In the present study, psilocybin impaired cognitive empathy in response to positive stimuli by decreasing the ability to correctly identify emotions. Consistent with these results, LSD decreased cognitive empathy in a previous study [37]. On the FERT, 2C-B had no significant effects on emotion recognition contrary to a previous study where 2C-B reduced the ability to recognize happiness [17]. However, there was a numerical decrease in the recognition of fearful faces, indicating trend effects that are similar to psilocybin. Psilocybin reduced emotion recognition, particularly the ability to recognize fearful faces. The effect of psilocybin on emotion recognition replicates findings from previous studies, in which 100 or 200 µg LSD reduced the recognition of fearful faces [37]. This lower recognition of negative emotions after the administration of serotonergic psychedelics parallels the finding of lower amygdala reactivity for psilocybin and LSD in other studies [43–46]. The moderate effects of 2C-B on emotional cognition indicate that although 2C-B induces strong psychedelic-type alterations of state of consciousness, the participants appeared to remain able to identify emotions correctly and may exhibit fewer thought impairments compared with psilocybin.

In the present study, we measured plasma levels of oxytocin and neurophysin I, which are implicated in empathy and emotion processing. MDMA increased levels of oxytocin and neurophysin I, which aligns with prior work [34, 42, 47], whereas 2C-B and psilocybin did not alter levels of circulating oxytocin or neurophysin I, indicating that their empathogen-like effects may not be critically mediated by oxytocin release. Consistent with these results, LSD increased emotional empathy but only minimally elevated circulating oxytocin [20, 36, 37].

In the present study, 2C-B exhibited dose-dependent but only relatively mild cardiovascular stimulation compared with MDMA and psilocybin, even at the highest dose of 30 mg 2C-B. A previous study suggested that 20 mg 2C-B induces comparable cardiovascular stimulation as 15 mg psilocybin [18]. Altogether, 2C-B exhibited a more favorable cardiovascular safety profile than 125 mg MDMA and a comparable or better cardiovascular safety profile compared with 25 mg psilocybin.

Acute effect durations of 20 and 30 mg 2C-B were similar to MDMA and shorter than psilocybin. The difference in the duration of action between psilocybin and 2C-B can be attributed to their pharmacokinetic profiles. The plasma half-lives of 2C-B and psilocybin are 1.3 and 2 h, respectively, which is consistent with their durations of action and previously reported pharmacokinetic data [18, 48, 49]. Importantly, the pharmacodynamic effects of all 2C-B doses, and psilocybin over time mimicked time courses of the respective substance concentrations in plasma. In contrast, MDMA exhibited a shorter acute effect duration despite its longer half-life of ~9 h. The shorter acute effect duration of 2C-B of 5 h versus 6 h for psilocybin may offer an advantage of 2C-B over psilocybin in clinical practice.

In the present study, effects of 2C-B were relatively well-blinded compared with MDMA and psilocybin. At the end of the study, the highest 30 mg dose of 2C-B was only correctly identified by 38% of the participants and was mistaken primarily for psilocybin by 38% of the participants, supporting their similar subjective effects.

The present study was the first to directly compare psilocybin and MDMA within the same study, and the participants received typical and therapeutically relevant doses. We found that both compounds induced comparable positive subjective effects, but psilocybin induced more negative effects and perceptual changes compared with MDMA. In contrast, a previous study that compared 125 mg MDMA and 100 µg LSD found marked differences between MDMA and LSD on all subscale ratings of the 5D-ASC, including positive effects [19]. This was also the case in another study that compared 100 mg MDMA and 100 µg LSD [50]. In the present study, we observed stronger subjective effect ratings for MDMA compared with prior studies, whereas ratings for psilocybin were comparable between the present and prior studies with psilocybin [21, 49, 51]. The present study design, which included three doses of 2C-B, may have resulted in higher ratings of MDMA’s effects, which were more similar to psilocybin. Thus, the present study showed that 125 mg MDMA may induce similar positive subjective effects as psilocybin. Acute positive effects of psilocybin predict therapeutic effects [52–54]. Remaining to be studied is whether acute positive effects of MDMA also correlate with the therapeutic response. Interestingly, the blinding assessment showed that MDMA was never mistaken for psilocybin, and psilocybin was never mistaken for MDMA, supporting clearly distinct subjective effects despite similar ratings for positive effects of these two substances as previously shown for MDMA and LSD [19, 50].

The present study has several strengths. We used three different doses of 2C-B in a statistically powerful within-subjects design. We compared both pharmacodynamic and pharmacokinetic effects of 2C-B with the prototypical entactogen MDMA, the prototypical classic serotonergic psychedelic psilocybin, and placebo under double-blind conditions in a highly controlled setting. The use of a six-arm design may also have improved blinding compared with other studies that used fewer arms.

The present study also has limitations. We included only healthy participants in a controlled setting. Thus, effects and safety profiles may look different in patients or in a recreational setting.

## Conclusion

In conclusion, the pharmacodynamic effects of 2C-B were partly MDMA-like and psilocybin-like, particularly at higher doses, indicating overlapping entactogenic and psychedelic effects. The effect duration of 2C-B was shorter than psilocybin, which aligns with its shorter plasma half-life. At the tested doses, 2C-B induced lower cardiovascular stimulation compared with MDMA and psilocybin. 2C-B also produced less psychological distress compared with psilocybin. These results may assist with dose finding for future 2C-B research and provide a direct comparison with standard doses of the prototypical compounds MDMA and psilocybin.

## Supplementary information

Supplemental Information

## Data Availability

Data is available from the corresponding authors upon reasonable request.
