# Supplementary material for: Acute dose-dependent effects of 4-bromo-2,5-dimethoxyphenethylamine (2C-B) compared with 3,4-methylenedioxymethamphetamine (MDMA) and psilocybin in a double-blind, placebo-controlled study in healthy participants
Source: Neuropsychopharmacology. 2026 Apr 28;51(8):1511–8. doi: 10.1038/s41386-026-02428-9 (PMC13291353; doi:10.1038/s41386-026-02428-9)
Supplement: Supplementary file 1 — Supplemental Information [file 41386_2026_2428_MOESM1_ESM.pdf]

Supplemental Information

Acute dose-dependent effects of 4-bromo-2,5-dimethoxyphenethylamine (2C-B) compared with 3,4-methylenedioxymethamphetamine (MDMA) and psilocybin in a double-blind, placebo-controlled study in healthy participants

Denis Arikci,[1,2] Joran Borgulya,[1,2] Isabelle Straumann,[1,2] Patrick Vizeli,[1,2] Dino Luethi,[1,2] Jan Thomann,[1,2] Deborah Rudin,[1,2] Ina Vukalovic[3,4] Anne Eckert,[3,4] Matthias E. Liechti[1,2], Friederike Holze[1,2]

[1]Clinical Pharmacology and Toxicology, Department of Biomedicine and Department of Clinical Research, University Hospital Basel; [2]Department of Pharmaceutical Sciences, University of Basel; [3]Psychiatric University Hospital, University of Basel, [4]Transfaculty Research Platform Molecular and Cognitive Neuroscience, University of Basel, Basel Switzerland.

Section 1 Supplementary Methods 2
Section 2 Supplementary Results 6
Section 3 Consort Flow-Chart 20
Section 4 References 21

Section 1 Supplementary Methods

Participants

Exclusion criteria were age < 25 years or > 65 years, pregnancy (urine pregnancy test at screening and before each test session), personal or family (first-degree relative) history of major psychiatric disorders (assessed by the Semi-structured Clinical Interview for *Diagnostic and Statistical Manual of Mental Disorders*, 5th edition, Axis I disorders by a trained psychiatrist), the use of medications that may interfere with the study medications (e.g., antidepressants, antipsychotics, and sedatives), chronic or acute physical illness (e.g., abnormal physical exam, electrocardiogram, or hematological and chemical blood analyses), tobacco smoking (> 10 cigarettes/day), lifetime prevalence of illicit drug use > 20 times (except for D^9-tetrahydrocannabinol), illicit drug use within the last 2 months, and illicit drug use during the study period (determined by urine drug tests). The participants were asked to consume no more than 20 standard alcoholic beverages/week and have no more than one alcoholic beverage on the day before the test sessions.

Study drugs

2C-B (ReseaChem, Burgdorf, Switzerland) was administered in opaque capsules that contained 5 mg 2C-B hydrochloride with an exact analytically confirmed 2C-B content of 4.99 mg. Psilocybin (ReseaChem, Burgdorf, Switzerland) was administered in opaque capsules that contained 5 mg psilocybin dihydrate with an exact analytically confirmed content of 4.96 mg. Racemic MDMA (ReseaChem, Burgdorf, Switzerland) was administered in opaque capsules that contained 25 mg MDMA hydrochloride with an exact analytically confirmed MDMA content of 23.8 mg. Placebo consisted of identical opaque capsules that were filled with mannitol. All capsules were produced according to Good Manufacturing Practice guidelines (Dr. Hysek AG, Biel, Switzerland). The subjects received six capsules in each session: (*i*) six placebo capsules, (*ii*) two 5 mg 2C-B capsules and four placebo capsules, (*iii*) four 5 mg 2C-B capsules and two placebo capsules, (*iv*) six 5 mg 2C-B capsules, (*v*) five 25 mg MDMA capsules and one placebo capsule, and (*vi*) five 5 mg psilocybin capsules and one placebo capsule.

Study procedures

The sessions were conducted in a calm hospital room. Only one research subject and one investigator were present during each test session. The test sessions began at 8:00 AM. A urine sample was taken to verify abstinence from drugs of abuse, and a urine pregnancy test was performed in women before each test session. The participants received a standardized breakfast (two croissants) and underwent baseline measurements. 2C-B (10, 20, or 30 mg), MDMA, psilocybin, or placebo was administered at 9:00 AM. Outcome measures were repeatedly assessed for 9 h. The participants remained under constant supervision during the acute effect phase (up to 6-8 h). The participants were released ~9 h after drug administration.

Visual Analog Scales (VASs)

Subjective effects were assessed repeatedly using visual analog scales (VASs) [1,2] one hour before and 0, 0.5, 1, 1.5, 2, 2.5, 3, 3.5, 4, 5, 6, 7, 8, and 9 h after drug administration. The VASs included ratings for “any drug effect”, “good drug effect”, “bad drug effect”, “drug high”, “anxiety”, “nausea”, “alteration of vision”, “alteration of hearing”, “sounds seem to influence what I see” (= audio-visual synesthesia), “altered perception of time” and “the boundaries between myself and my surroundings seem to blur” (= ego-dissolution) that were presented as 100-mm horizontal lines (0-100%), marked from “not at all” on the left to “extremely” on the right [1,3]. Further VASs included “trust”, “open”, “closeness to others”, “I want to be alone”, and “I want to be with others”. These VASs were bidirectional and marked with “normal” in the middle at 0 mm and “not at all” (-50 mm) on the left and “extremely” (50 mm) on the right. VAS “alteration of vision”, “sounds seem to influence what I see”, “alteration of sense of time” and “ego dissolution” have previously been shown to be sensitive to different psychedelics and MDMA [4-6]. The bidirectional VASs were used to investigate changes in interpersonal relations to assess potential entactogenic effects. The VAS can be completed relatively rapidly and easily by the participant during the acute psychoactive experience and allows for a valid real-time assessment of the drug effects over time. The VASs are sensitive and relatively simple measures. VASs were assessed each time when blood drug concentrations were measured.

Adjective Mood Rating Scale (AMRS)

The Adjective Mood Rating Scale (AMRS) [7] was used before, and 3, 6, and 9 h after drug administration. The AMRS is a validated 60-item Likert mood rating scale mainly used in Europe and consists of subscales including ratings on “well-being”, “sensitivity”, “extraversion”, “introversion”, “activity”, “inactivity”, “concentration” and “fear”. It is suitable for repeated assessments of mood states. The short German EWL60S version was used [7]. The completion of the ratings during the acute effects of psychedelic substances is possible but challenging for the participants because it takes several minutes to complete the questionnaire. The scale was used in paper and pencil version, but items may also be read to the participant by the investigator and rated verbally by the participant. The AMRS was included as a secondary measure because it could be considered a better validated measure of mood states and produces more defined ratings than the VASs and to support findings on the VASs (i.e., AMRS well-being could be considered similar to VAS good drug effects; AMRS fear could be considered similar to VAS anxiety; etc.).

5 Dimension of Altered States of Consciousness (5D-ASC) scale

The 5 Dimensions of Altered States of Consciousness (5D-ASC) scale [8,9] was used as the primary outcome measure for psychedelic-like effects and was completed 9 h after drug administration to retrospectively rate peak drug effects. The German 5D-ASC scale measures altered states of consciousness and contains 94 items (visual analog scales). The instrument consists of five subscales/dimensions [8] and 11 lower-order scales (42-items). We herein report the lower-order scales according to the recent revalidation (3D-ASCr scale) of the 5D-(11)-ASC [10] where 3 higher order

dimensions “Positive effects”, “Distressing effects”, and “Perceptual effects” consist of the 11 lower-order scales described by Studerus et al. [9]. The “Positive effects” scale includes the lower order scales “experience of unity,” “spiritual experience,” “blissful state,” “insightfulness,” “changed meaning of percepts”, and “disembodiment.” The “Distressing effects” scale includes the two lower order scales “impaired control of cognition” and “anxiety.” The “Perceptual effects” scale comprises the lower-order scales “complex imagery,” “elementary imagery,” and “audio-visual synesthesia”.

To facilitate comparison with older studies, we also show the original dimensions “Oceanic Boundlessness” (27 items), “Anxious Ego Dissolution” (21 items), Visionary Restructuralization” (18 items), “Auditory Alterations” (15 items) and “Reduction of Vigilance” (12 items) which can be summarized into the 5D-ASC total score. The total 3D-ASC score is the total of the three main dimensions “Oceanic Boundlessness”, “Anxious Ego-Dissolution”, and “Visionary Restructuralization” and can be used as a measure of the overall intensity of the alteration of the mind [11]. The scale is well-validated in German [8,10] and many other languages and widely used to characterize the subjective effects of various psychedelic drugs. In particular, the scale has been used by most research groups to psychometrically assess LSD, psilocybin and MDMA effects [1,2,6,10,12-16]. Furthermore, acute ratings on the 5D-ASC after administration of psilocybin and LSD have been used to predict long-term effects of psychedelic treatments in patients [17-19]. Ratings on the 5D-ASC have been shown to closely correlate with ratings on the Mystical Effects Questionnaire (MEQ, see below) [11] which is primarily used by research groups in the US [18].

Psychedelic Experience Scale (PES) and Mystical Effects Questionnaire (MEQ)

Mystical experiences were assessed 9 h after drug administration using the Psychedelic Experience Questionnaire/Scale (PES) [20] that represents a revalidation of the original 100-item States of Consciousness Questionnaire (SOCQ) [11,21] and includes the 43-item Mystical Effects Questionnaire (MEQ43) [21], the 30-item Mystical Effects Questionnaire (MEQ30) [22], and the 40-item Mystical Effects Questionnaire (MEQ40) [20]. The MEQ30 subscales are “mystical”, “positive mood”, “transcendence of time/space”, and “ineffability” and their total provides the MEQ30 total score. Ten more items allow for the derivation of the additional subscales “paradoxicality” and “connectedness” (40-item MEQ40). Eight more items allow to derive the additional “visual experience” and “distressing experience” subscales that together with all other subscales for the PES subscales (48 items from the 100-item SOCQ. Note that the full 100-item questionnaire was completed by the participants and only 48 items are needed to derive the validate subscales [20]. The published German version was used [20].The MEQ has been used in numerous experimental and therapeutic trials with psilocybin [18,21,23-29]. The MEQ has also been used in many experimental trials with LSD and MDMA [1,2,5,6,30,31].

Multifaceted Empathy Test (MET)

The MET is a reliable and valid task used to assess cognitive and emotional aspects of empathy [32] and has previously been shown to be sensitive to MDMA [3,33], LSD [13,34], and psilocybin [35]. The computer-assisted test consists of 40 photographs showing individuals in emotionally charged situations.

To assess cognitive empathy, participants were asked to choose one of four adjectives best describing a person's emotional state shown in a picture. Cognitive empathy was defined as the percentage of correct responses. Emotional empathy is assessed as explicit and implicit emotional empathy. To measure explicit emotional empathy, which can be regarded as an equivalent to empathic concern, subjects were asked to indicate how strongly they were feeling for an individual shown in each scene on a 9-point Likert scale (1 = not at all, 9 = very strongly). To measure implicit emotional empathy, subjects were asked to rate how aroused they felt by each scene on a 9 –point Likert scale (1 = very calm, 9 = very aroused). The implicit empathy rating provides an inherent additional assessment of emotional empathy, which is considered to reduce the likelihood of socially desirable answers. The three aspects of empathy were each assessed with 20 stimuli with positive valence and 20 stimuli with negative valence, resulting in a total of 120 trials. The MET was performed 3 h after drug administration.

Facial Emotion Recognition Task

The Facial Emotion Recognition Task (FERT) is a widely used task to assess the ability to recognize emotions from facial expressions. The task has previously been shown to be sensitive to psychedelics [13], MDMA [3,33,36,37], and stimulants [33,37,38]. The task included 10 neutral faces and 160 faces that expressed one of four basic emotions (i.e., happiness, sadness, anger, and fear), with pictures morphed between 0% (neutral) and 100% in 10% steps. Two female and two male pictures were used for each of the four emotions. The stimuli were presented in random order for 500 ms and then were replaced by the rating screen where participants had to indicate the correct emotion. The main outcome measure was accuracy (proportion correct). The FERT was performed right after the MET ~3 h after drug administration.

Sample size calculation

Power analysis was performed with PASS®, Hintze J. Kaysville, Utah, US. A difference of 15% in the primary measure (5D-ASC total score) is considered clinically meaningful. A sample size of 16 achieves 80% power to detect a difference of 15% between the null hypothesis mean of 100% and the alternative hypothesis mean of 85% with an estimated standard deviation of 20% and with a significance level (alpha) of 0.05 using a two-sided one-sample t-test. The study will be adequately powered for the primary endpoint with a sample size > 16. A minimal sample size of 20 accounts for the additional secondary endpoints although these analyses will remain more exploratory and/or confirmatory of the primary endpoint findings but using alternative and additional measures. Based on our experience with a similar study we assume a screening-failure rate of 25% and we expect to screen 36 subjects to include 24 in the study. A total of 24 participants is planned for the study, consisting of 12 males and 12 females.

Section 2 Supplementary Results

Supplementary Figures

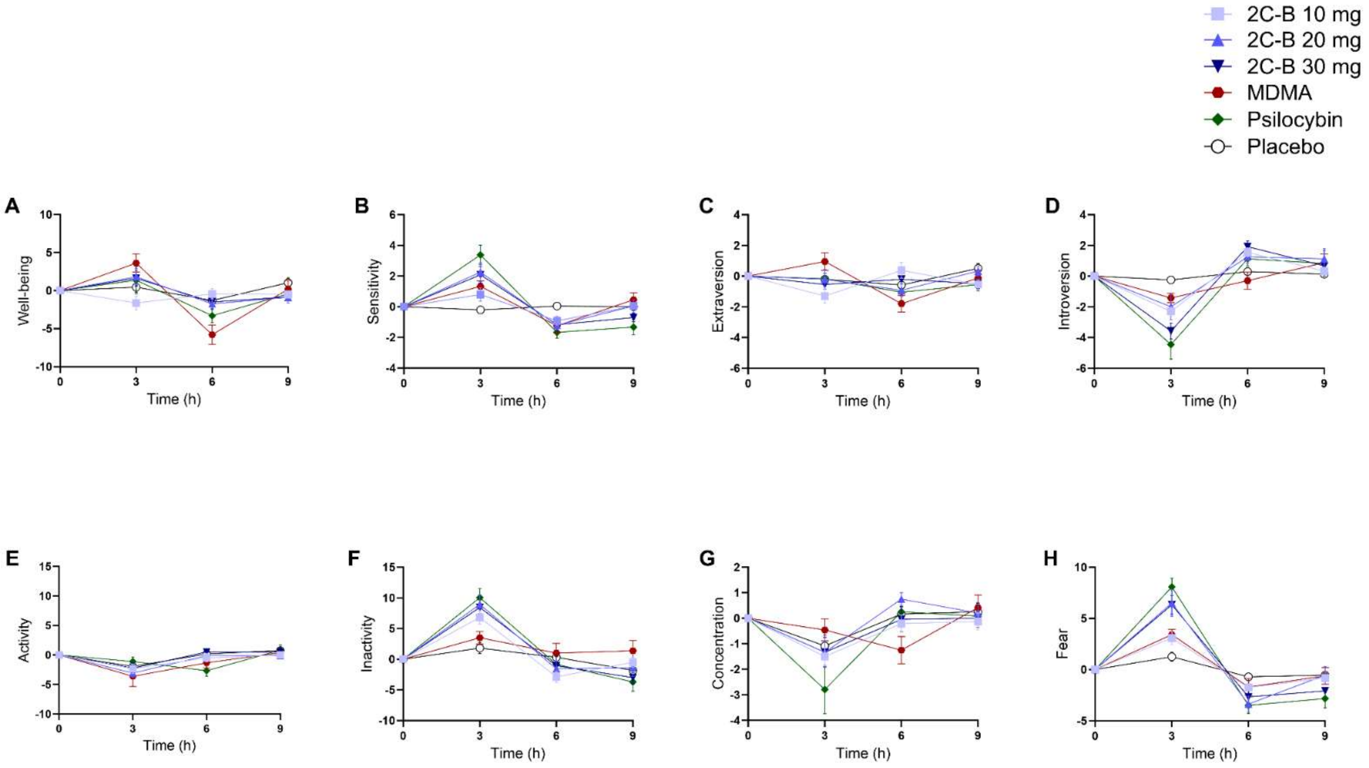

Figure S1. Subjective mood over time on the Adjective Mood Rating Scale (AMRS). All data are expressed as mean ± SEM changes from baseline in 24 participants. The corresponding maximal effects and statistics are shown in **Supplementary Table S3.**

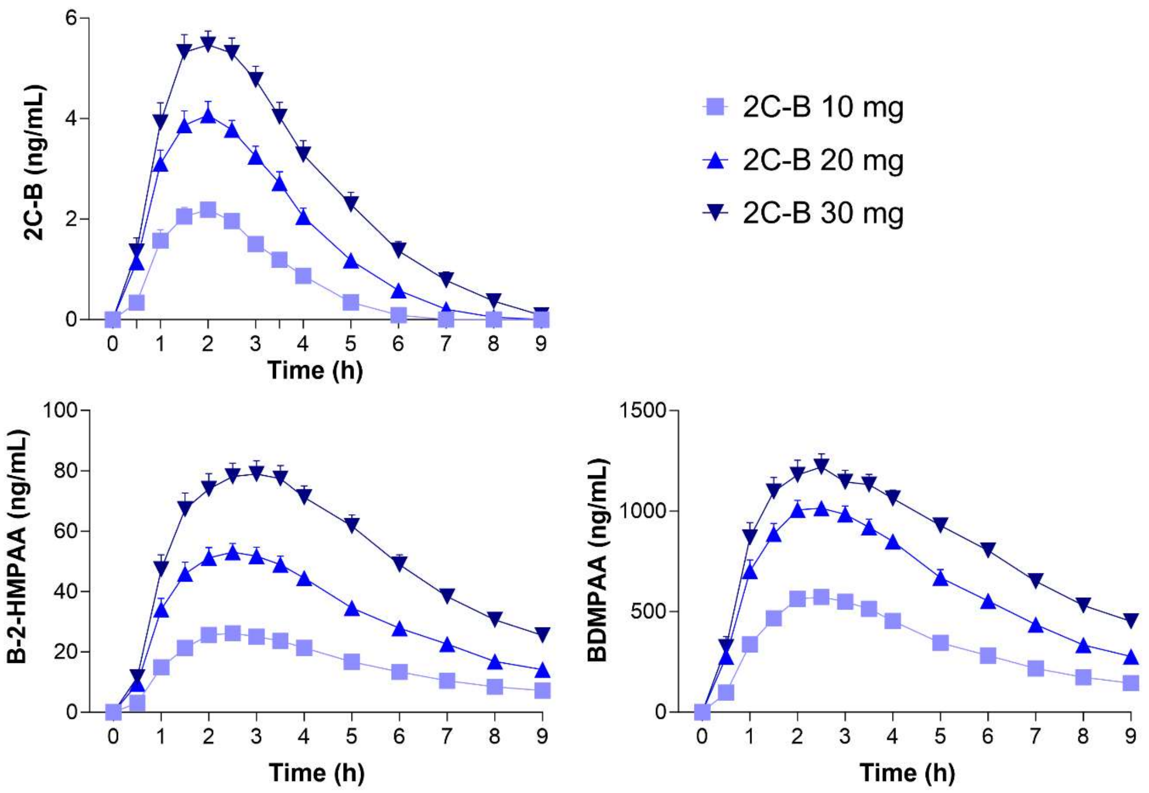

Figure S2. Plasma concentrations of 10, 20, and 30 mg 2C-B and its main metabolites B-2-HMPAA (4-bromo-2-hydroxy-5-methoxyphenylacetic acid) and BDMPAA (4-bromo-2,5-dimethoxyphenylacetic acid) over time. The data are expressed as mean ± SEM in 24 participants. 2C-B was administered at t = 0 h. The corresponding pharmacokinetic parameters are shown in **Supplementary Table S7**.

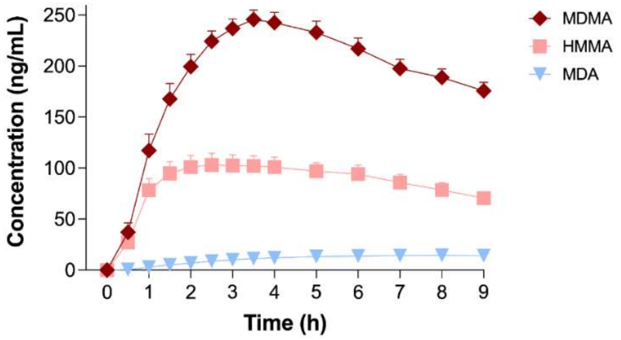

Figure S3. Plasma concentrations of MDMA and its main metabolites HMMA (4-Hydroxy-3-methoxymethamphetamin) and MDA (3,4-methylenedioxyamphetamine)
The data are expressed as mean ± SEM in 24 participants. The 125 mg dose of MDMA was administered at t = 0 h. The corresponding pharmacokinetic parameters are shown in **Supplementary Table S7.**

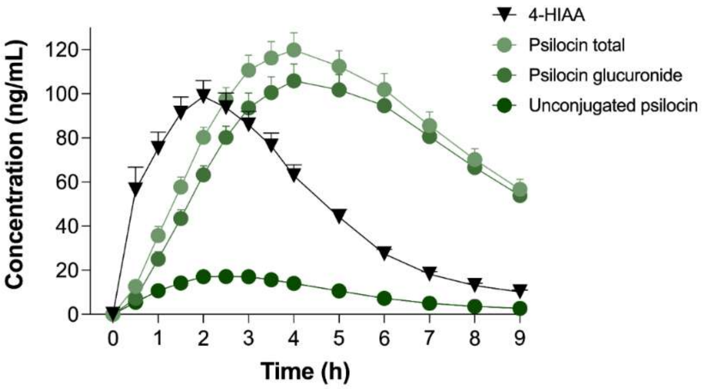

Figure S4. Plasma concentrations of the psychoactive unconjugated psilocin, inactive psilocin glucuronide, total psilocin, and the inactive metabolite 4-HIAA (4-hydroxindole-3-acetic acid). The data are expressed as mean ± SEM in 24 participants. The 25 mg dose of psilocybin was administered at t = 0 h. The corresponding pharmacokinetic parameters are shown in **Supplementary Table S7**.

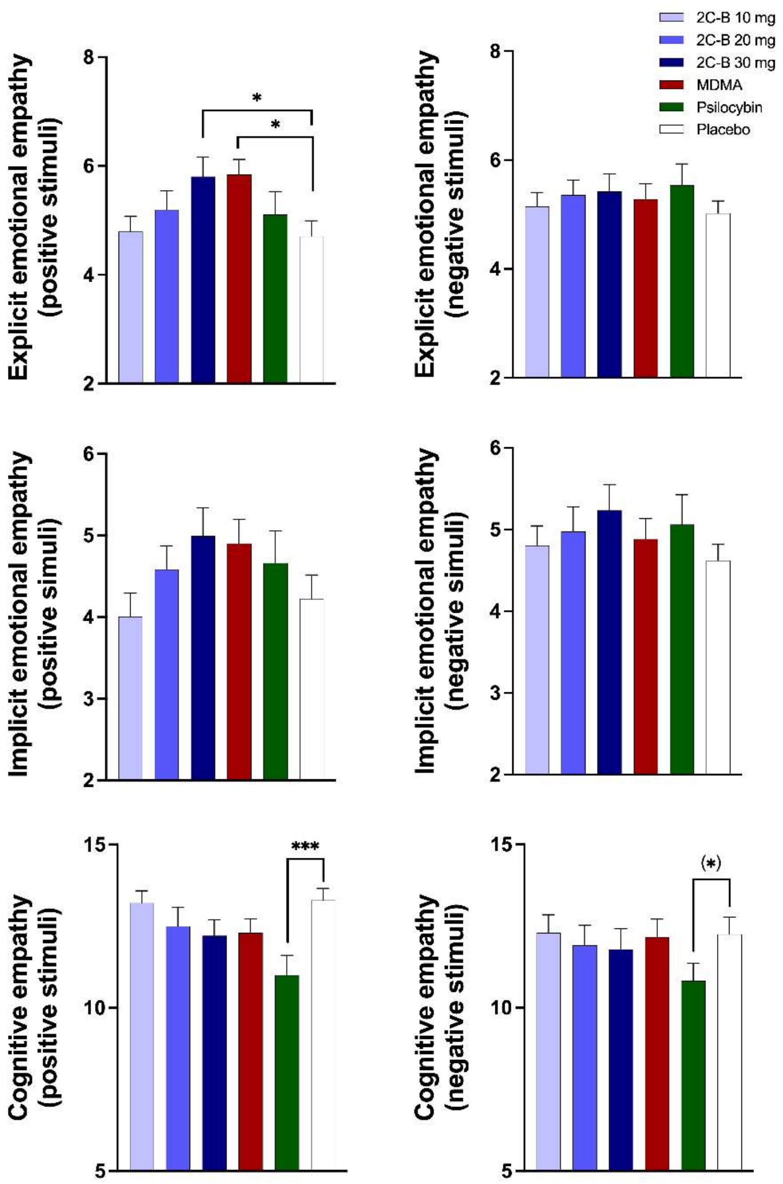

Figure S5. Cognitive empathy, and explicit and implicit emotional empathy assessed with the multifaceted empathy test (MET). The MET was performed 3 h after substance administration. The 30 mg dose of 2C-B and MDMA increased explicit emotional empathy of positive stimuli compared with placebo. Psilocybin decreased cognitive empathy to positive stimuli. The data are presented as mean ± SEM in 24 participants (23 participants in the psilocybin condition). Differences compared to placebo are marked as $^{(*)}$ $p < 0.1$ *$p < 0.05$, ***$p < 0.001$.

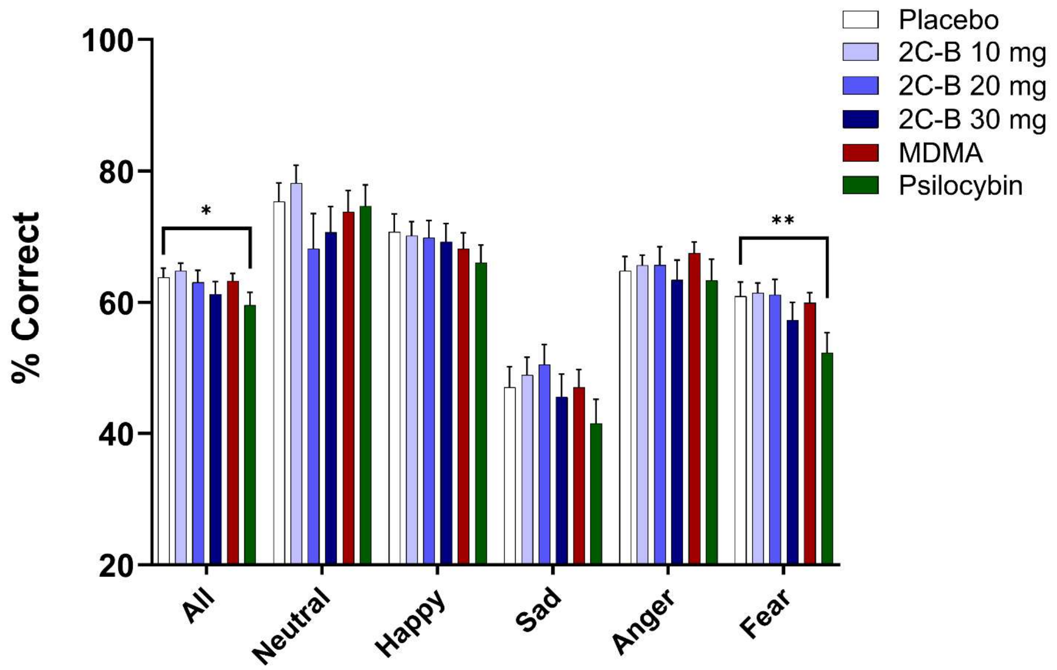

Figure S6. Facial Emotion Recognition Task (FERT). The FERT was performed after the MET i.e. 3 h after substance administration. Psilocybin reduced overall correct identification of emotions, mostly driven by impaired recognition of fearful faces. The data are presented as mean ± SEM in 24 participants (23 participants in the psilocybin condition). Significant differences compared to placebo are marked as *$p < 0.05$, **$p < 0.01$.

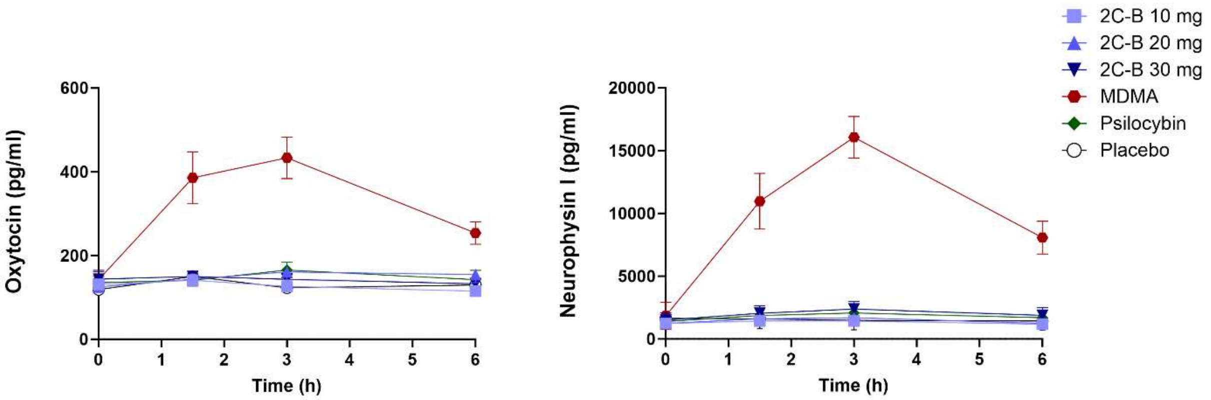

Figure S7. Plasma concentrations of oxytocin and its carrier protein neurophysin I after administration of 10 mg, 20 mg and 30 mg 2C-B, MDMA, psilocybin, and placebo. Only MDMA increased oxytocin and neurophysin I plasma concentrations compared with placebo. The data are expressed as mean ± SEM in 24 participants. Drug or placebo was administered at t = 0 h.

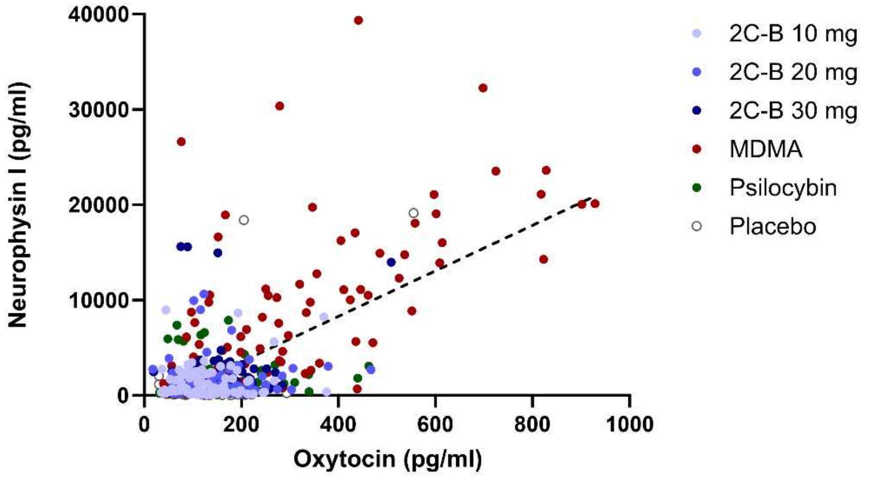

Figure S8. Correlation between oxytocin and neurophysin I plasma concentrations after administration of 10 mg, 20 mg and 30 mg 2C-B, MDMA, psilocybin, and placebo. Oxytocin concentrations significantly correlated with neurophysin I concentrations ($r_p = 0.63$, $p < 0.001$).

Supplementary Tables

Table S1. Sample characteristics and substance use history (N=24)

Demographics	*N* =	Mean ± SD (Range)
N (male/female)	24 (12/12)	
Age (years)		36 ± 9.0 (25 - 52)
Weight (kg)		68 ± 12 (51 - 91)
Height (cm)		175 ± 11 (158 - 197)
Body Mass index (kg/m2)		22 ± 2.5 (18 - 29)
**Current and Lifetime Drug Use**	***N* =**	**Mean ± SD (Range)**
Caffeine (servings per day)	23	2.3 ± 1.3 (1 - 5)
Nicotine	6	
Smokers (cigarettes per day)	5	2.2 ± 1.6 (1 - 5)
Snus (snus per day)	1	1
Alcohol (drinks per week)	23	3.5 ± 3.3 (1 - 15)
Cannabis (lifetime use)	22	69 ± 114 (1 - 400)
MDMA (lifetime use)	11	3.3 ± 2.0 (1 - 6)
Psychedelics (lifetime use)	13	5.7± 4.2 (1 - 11)
Ketamine (lifetime use)	2	2.3 ± 2.3 (1 - 5)
Stimulants (lifetime use)	6	5.3 ± 6.7 (1 - 20)
Opioids (lifetime use)	0	0
Other drugs (lifetime use)	1	1
Any illicit drug use (except for caffeine, nicotine, alcohol and cannabis)	20	6.3 ± 5.9 (1 - 20)

N= number of participants

Table S2. Mean values and statistics for the acute subjective, autonomic and side effects of of 10, 20 and 30 mg 2C-B, MDMA, psilocybin, and placebo

		Placebo (mean ± SEM)	2C-B 10 mg (mean ± SEM)	2C-B 20 mg (mean ± SEM)	2C-B 30 mg (mean ± SEM)	MDMA (mean ± SEM)	Psilocybin (mean ± SEM)	$F_{5,115}$	η_p^2	P=	Pla - 2C-B 10 mg	Pla - 2C-B 20 mg	Pla - 2C-B 30 mg	Pla - MDMA	Pla - Psilo	2C-B 10 mg - 20 mg	2C-B 10 mg - 30 mg	2C-B 10 mg - MDMA	2C-B 10 mg - Psilo	2C-B 20 mg - 30 mg	2C-B 20 mg - MDMA	2C-B 20 mg - Psilo	2C-B 30 mg - MDMA	2C-B 30 mg - Psilo	MDMA - Psilo
Visual Analogue Scale (VAS, %max)																									
Unidirectional Scales (0-100)																									
Any drug effect	ΔE_{max}	7.4 ± 2.5	27 ± 4.1	56 ± 5.2	72 ± 4.5	70 ± 4.4	88 ± 3.6	77.41	0.77	<0.001	0.71**	1.95***	2.55***	2.48***	4.13***	1.02***	2.03***	1.7***	2.82***	0.7*	0.51(*)	1.55***	0.07	0.73*	0.71**
Good drug effect	ΔE_{max}	10 ± 4.2	29 ± 5.4	60 ± 5.9	68 ± 4.7	78 ± 4.8	84 ± 4.0	50.13	0.69	<0.001	0.47*	1.61***	2.03***	2.16***	2.87***	0.98***	1.49***	1.65***	2.04***	0.37	0.65*	0.94***	0.41	0.57(*)	0.25
Bad drug effect	ΔE_{max}	0 ± 0	2.0 ± 3.2	7.4 ± 2.4	9.8 ± 2.5	7.7 ± 3.3	19 ± 5.7	5.04	0.18	<0.001	0.63	0.64	0.81	0.48	0.67***	0.47	0.58	0.33	0.59**	0.15	0.02	0.39(*)	0.15	0.3	0.38(*)
Drug high	ΔE_{max}	6.8 ± 4.2	17 ± 4.8	45 ± 6.4	53 ± 6.1	67 ± 6.2	64 ± 7.6	26.53	0.54	<0.001	0.3	1.25***	1.57***	1.79***	1.45***	0.81***	1.21***	1.68***	1.36***	0.22	0.6*	0.56(*)	0.49	0.29	0.11
Anxiety	ΔE_{max}	0.3 ± 0.2	0.8 ± 0.6	5.6 ± 3.3	3.8 ± 1.6	3.7 ± 1.5	13 ± 4.5	4.67	0.17	<0.001	0.17	0.32	0.45	0.53	0.56***	0.33	0.52	0.37	0.59**	0.17	0.1	0.56	0.01	0.52*	0.39*
Nausea	ΔE_{max}	0.5 ± 0.4	3.2 ± 1.4	11 ± 3.1	13 ± 3.9	6.5 ± 2.9	18 ± 5.1	5.64	0.20	<0.001	0.41	0.69	0.67*	0.43	0.71***	0.52	0.52	0.25	0.63**	0.14	0.27	0.39	0.32	0.2	0.44*
Visual alteration	ΔE_{max}	1.5 ± 0.7	12 ± 3.0	40 ± 6.7	57 ± 6.1	25 ± 5.4	77 ± 5.7	46.25	0.67	<0.001	0.67	1.2***	1.86***	0.93**	2.74***	0.93***	1.48***	0.44	2.61***	0.52*	0.37	1.46***	1.17***	0.78*	1.76***
Auditory alteration	ΔE_{max}	3.2 ± 1.6	12 ± 3.6	32 ± 6.3	41 ± 5.9	30 ± 6.0	59 ± 6.9	23.92	0.51	<0.001	0.44	1.05***	1.39***	0.93***	1.72***	0.62**	0.95***	0.67*	1.71***	0.25	0.09	0.93***	0.42	0.68*	1.36***
Audio-visual synaesthesia	ΔE_{max}	2.4 ± 1.5	12 ± 4.7	35 ± 7.4	47 ± 6.3	25 ± 6.0	69 ± 5.8	27.95	0.55	<0.001	0.4	0.97***	1.53***	0.79**	2.48***	0.65**	1.01***	0.41	2.13***	0.29	0.23	1.17***	0.68*	0.81**	1.64***
Alteration of sense of time	ΔE_{max}	3.5 ± 2.6	13 ± 4.2	43 ± 7.5	59 ± 6.4	42 ± 5.9	75 ± 6.1	37.97	0.62	<0.001	0.38	1.13***	1.88***	1.45***	2.4***	0.86***	1.39***	0.94***	1.97***	0.52(*)	0.04	1.22***	0.54(*)	0.67	1.12***
Ego dissolution	ΔE_{max}	4.3 ± 4.2	8.8 ± 3.0	37 ± 7.3	51 ± 7.7	31 ± 5.8	71 ± 6.5	30.24	0.57	<0.001	0.17	0.97***	1.25***	1.08***	1.96***	0.89***	1.2***	0.85**	2.22***	0.34	0.18	1.19***	0.58*	0.7*	1.25***
Bidirectional Scales (-50 to 50)																									
Trust	ΔE_{max}	3.1 ± 2.1	6.1 ± 1.9	18 ± 3.6	20 ± 3.6	31 ± 3.0	25 ± 4.2	19.47	0.46	<0.001	0.2	0.85***	0.99***	1.84***	1.09***	0.71*	0.83***	1.89***	0.93***	0.17	0.87**	0.38	0.67*	0.22	0.41
	ΔE_{min}	0 ± 0	- 0.4 ± 0.2	- 2.1 ± 2.1	- 2.0 ± 1.3	- 0.2 ± 0.2	- 4.4 ± 2.3	1.80	0.07	0.117	0.32	0.2	0.3	0.2	0.38	0.17	0.24	0.15	0.35	0.02	0.19	0.21	0.27	0.22	0.36
Open	ΔE_{min}	2.7 ± 1.6	7.6 ± 2.4	19 ± 3.7	22 ± 3.5	32 ± 2.9	28 ± 4.0	22.04	0.49	<0.001	0.32	0.94***	1.23***	2.11***	1.34***	0.67*	0.79***	1.74***	1.03***	0.19	0.88**	0.46	0.59(*)	0.27	0.34
	ΔE_{min}	- 1.4 ± 0.8	- 0.4 ± 0.3	- 3.8 ± 2.0	- 2.9 ± 1.4	- 2.7 ± 1.3	- 6.9 ± 2.9	2.70	0.11	0.024	0.19	0.42	0.28	0.28	0.43*	0.35	0.36	0.34	0.47*	0.13	0.14	0.23	0.03	0.3	0.32
Closeness to others	ΔE_{max}	3.3 ± 2.1	3.1 ± 1.1	17 ± 3.3	20 ± 3.7	28 ± 3.1	27 ± 3.9	21.77	0.49	<0.001	0.02	0.92**	0.91***	1.59***	1.29***	0.92***	0.94***	1.75***	1.34***	0.17	0.72*	0.57*	0.47	0.38	0.07
	ΔE_{min}	- 0.3 ± 0.3	- 2.2 ± 1.1	- 4.5 ± 2.4	- 6.0 ± 2.2	- 5.5 ± 2.3	- 13 ± 3.3	5.48	0.19	<0.001	0.43	0.38	0.51	0.43	0.79***	0.2	0.34	0.24	0.67**	0.13	0.08	0.63*	0.05	0.41	0.54(*)
I want to be alone	ΔE_{max}	2.2 ± 1.6	3.8 ± 1.4	9.8 ± 3.2	12 ± 3.0	8.6 ± 2.6	17 ± 3.5	8.06	0.26	<0.001	0.16	0.59(*)	0.8**	0.7	1***	0.41	0.61*	0.35	0.87***	0.21	0.09	0.51(*)	0.36	0.35	0.5*
	ΔE_{min}	- 1.9 ± 1.0	- 4.6 ± 1.9	- 23 ± 4.2	- 19 ± 4.0	- 28 ± 4.3	- 23 ± 4.4	12.73	0.36	<0.001	0.26	1.08***	0.97**	1.33***	1.02***	0.92***	0.71*	1.04***	0.95***	0.22	0.21	0.02	0.39	0.21	0.21
I want to be with others	ΔE_{max}	3.0 ± 1.7	3.0 ± 1.1	14 ± 3.2	15 ± 3.4	19 ± 3.5	23 ± 4.2	10.85	0.32	<0.001	0	0.81*	0.76*	1.04***	1.03***	0.73*	0.73*	1.03***	1.03***	0.02	0.26	0.4	0.21	0.4	0.18
	ΔE_{min}	- 0.6 ± 0.4	- 4.3 ± 1.7	- 12 ± 3.8	- 12 ± 3.0	- 11 ± 2.6	- 22 ± 4.0	10.18	0.31	<0.001	0.48	0.62*	0.76*	0.85*	1.13***	0.43	0.58	0.49	1.02***	0.02	0.03	0.71*	0.02	0.61*	0.6*
Autonomic Effects																									
Systolic blood pressure (mmHg)	E_{max}	120 ± 2.0	124 ± 1.8	127 ± 1.7	128 ± 1.9	145 ± 2.6	142 ± 2.4	72.63	0.76	<0.001	0.51	1.31**	1.5**	2.36***	2.51***	0.4	0.45	2.31***	2.14***	0.14	1.8***	1.88***	1.72***	1.71***	0.28
Diastolic blood pressure (mmHg)	E_{max}	73 ± 1.4	77 ± 1.5	78 ± 1.3	79 ± 1.4	87 ± 1.7	89 ± 1.8	50.11	0.66	<0.001	0.4	0.85*	0.92*	1.6***	2.09***	0.34	0.36	1.55***	1.94***	0.07	1.25***	1.64***	1.1***	1.68***	0.36
Mean arterial pressure (mmHg)	E_{max}	88 ± 1.5	92 ± 1.5	94 ± 1.3	95 ± 1.4	106 ± 1.9	106 ± 1.9	64.94	0.72	<0.001	0.47	1.05**	1.12**	1.96***	2.43***	0.43	0.41	1.96***	2.17***	0.07	1.58***	1.92***	1.38***	1.91***	0.07
Heart rate (beats/min)	E_{max}	70 ± 1.8	72 ± 2.4	73 ± 2.3	76 ± 2.5	86 ± 3.6	76 ± 2.5	19.50	0.46	<0.001	0.26	0.6	0.76**	1.22***	0.58*	0.26	0.69	1.33***	0.56	0.43	1.12***	0.32	1.09***	0.02	0.91***
Rate pressure product (mmHg x bpm)	E_{max}	8188 ± 234	8454 ± 309	9056 ± 297	9358 ± 366	12159 ± 575	10221 ± 425	43.72	0.66	<0.001	0.25	0.97(*)	1**	1.73***	1.2***	0.67	0.91*	1.79***	1.3***	0.34	1.5***	0.94**	1.56***	0.73(*)	0.94***
Body temperature (°C)	E_{max}	37.2 ± 0.1	37.3 ± 0.1	37.5 ± 0.1	37.5 ± 0.1	37.5 ± 0.1	37.9 ± 0.1	18.84	0.45	<0.001	0.06	0.83*	0.79*	0.55(*)	1.51***	0.54*	0.72*	0.46	1.53***	0	0.17	0.79***	0.17	1.08***	1.17***
EKG QTc (ms)	Δ2h	0.4 ± 1.7	- 0.4 ± 1.6	- 3.4 ± 2.0	- 0.1 ± 2.3	- 0.9 ± 2.4	- 4.3 ± 2.3	0.83	0.04	NS	0.1	0.31	0.04	0.12	0.28	0.26	0.02	0.04	0.27	0.23	0.14	0.07	0.04	0.29	0.18
List of Complaints (LC Score)																									
Acute adverse effects	0-9 h	1.5 ± 0.6	5.2 ± 1.0	8.5 ± 1.4	9.5 ± 1.5	8.1 ± 1.4	11 ± 1.9	11.15	0.33	<0.001	0.63	1.15***	1.12***	0.99***	1.21***	0.45	0.64*	0.38	0.71***	0.21	0.05	0.38	0.2	0.25	0.39
Adverse effects	E_{max}	2.8 ± 0.7	6.3 ± 1.0	9.3 ± 1.5	11 ± 1.5	9.1 ± 1.5	12 ± 1.9	10.45	0.31	<0.001	0.59	1.01***	1.07***	0.88***	1.19***	0.41	0.65(*)	0.35	0.7**	0.23	0.02	0.41	0.2	0.26	0.39

Posthoc comparisons display Cohen's d and p-value: (*)P<0.1, *P<0.05, **P<0.01, ***P<0.001; η_p^2, partial eta-squared; NS, not significant; ΔE_{max}, maximal effect difference from baseline; ΔE_{min}, minimal effect difference from baseline; N=24

Table S3. Mean values and statistics for the acute subjective effects of 10, 20 and 30 mg 2C-B, MDMA, psilocybin and placebo on the Adjective Mood Rating Scale (AMRS)

		Placebo (mean ± SEM)	2C-B 10 mg (mean ± SEM)	2C-B 20 mg (mean ± SEM)	2C-B 30 mg (mean ± SEM)	MDMA (mean ± SEM)	Psilocybin (mean ± SEM)	$F_{5, 115}$	η_p^2	P=	Pla - 2C-B 10 mg	Pla - 2C-B 20 mg	Pla - 2C-B 30 mg	Pla - MDMA	Pla - Psilo	2C-B 10 mg - 20 mg	2C-B 10 mg - 30 mg	2C-B 10 mg - MDMA	2C-B 10 mg - Psilo	2C-B 20 mg - 30 mg	2C-B 20 mg - MDMA	2C-B 20 mg - Psilo	2C-B 30 mg - MDMA	2C-B 30 mg - Psilo	MDMA - Psilo
Adjective Mood Rating Scale (AMRS, score)																									
General well-being	ΔE_{max}	1.2 ± 0.8	-0.3 ± 0.62	1.8 ± 0.7	2.2 ± 1	4.3 ± 1.1	3.0 ± 1	4.10	0.15	0.002	0.32	0.16	0.28	0.45(*)	0.31	0.48	0.52	1.1***	0.71*	0.09	0.36	0.23	0.28	0.15	0.2
	ΔE_{min}	-2.1 ± 0.6	-2.9 ± 0.7	-3.3 ± 0.7	-1.8 ± 0.8	-2.7 ± 0.9	-2.4 ± 0.9	0.82	0.03	0.538	0.21	0.3	0.06	0.12	0.07	0.09	0.29	0.09	0.13	0.31	0.13	0.19	0.2	0.17	0.08
Sensitivity	ΔE_{max}	-0.1 ± 0.17	0.8 ± 0.44	2.0 ± 0.5	2.5 ± 0.5	1.8 ± 0.3	3.7 ± 0.6	10.80	0.32	<0.001	0.4	0.78**	0.96***	1.36*	1.22***	0.45	0.58*	0.46	0.76***	0.17	0.07	0.55*	0.22	0.39	0.64*
	ΔE_{min}	-0.3 ± 0.15	-0.5 ± 0.26	0.2 ± 0.26	-0.1 ± 0.33	0 ± 0.25	0.9 ± 0.30	3.90	0.15	0.003	0.14	0.27	0.12	0.2	0.68*	0.38	0.3	0.3	0.72**	0.14	0.12	0.44	0.04	0.52(*)	0.58
Extraversion	ΔE_{max}	0.8 ± 0.34	0.1 ± 0.44	-0.2 ± 0.29	0.5 ± 0.46	1.4 ± 0.5	1 ± 0.53	2.20	0.09	0.055	0.24	0.5	0.08	0.23	0.07	0.12	0.15	0.53	0.27	0.25	0.68*	0.46	0.3	0.14	0.2
	ΔE_{min}	-1.4 ± 0.3	-2.1 ± 0.4	-2.2 ± 0.4	-2.1 ± 0.4	-1.7 ± 0.4	-1.6 ± 0.4	0.97	0.04	0.438	0.31	0.35	0.3	0.14	0.11	0.05	0.02	0.2	0.19	0.03	0.21	0.26	0.2	0.23	0.04
Introversion	ΔE_{max}	0.5 ± 0.26	2.2 ± 0.3	2.9 ± 0.5	3.9 ± 0.5	2 ± 0.3	4.3 ± 0.5	14.30	0.38	<0.001	0.95*	0.82***	1.57***	0.71(*)	1.34***	0.24	0.65*	0.15	0.78**	0.34	0.34	0.52(*)	0.74**	0.18	0.9***
	ΔE_{min}	-0.2 ± 0.11	0.1 ± 0.21	0.6 ± 0.16	0.7 ± 0.32	0.4 ± 0.21	0.8 ± 0.30	3.30	0.13	0.007	0.34	0.78(*)	0.62*	0.55	0.69*	0.39	0.38	0.26	0.36	0.02	0.19	0.11	0.18	0.06	0.24
Activity	ΔE_{max}	0.2 ± 0.32	-0.8 ± 0.45	-0.7 ± 0.38	-0.2 ± 0.45	-0.1 ± 0.49	0.1 ± 0.58	1.10	0.05	0.353	0.4	0.47	0.15	0.13	0.06	0.05	0.23	0.3	0.29	0.17	0.19	0.27	0.02	0.08	0.07
	ΔE_{min}	-1.9 ± 0.4	-1.9 ± 0.4	-2.1 ± 0.3	-2.1 ± 0.4	-2.5 ± 0.5	-2.5 ± 0.4	0.61	0.03	0.693	0	0.1	0.09	0.22	0.25	0.09	0.07	0.24	0.21	0.02	0.15	0.18	0.21	0.23	0
Inactivity	ΔE_{max}	1.6 ± 0.3	2.8 ± 0.5	4.0 ± 0.5	3.8 ± 0.6	3.4 ± 0.6	4.8 ± 0.6	6.70	0.23	<0.001	0.46	0.99**	0.67**	0.58*	1***	0.45	0.33	0.16	0.65*	0.11	0.19	0.36	0.1	0.44	0.46
	ΔE_{min}	0 ± 0.21	0.9 ± 0.37	1.5 ± 0.3	0.9 ± 0.39	0.4 ± 0.31	2.1 ± 0.5	6.50	0.22	<0.001	0.6	0.99**	0.48	0.24	0.99***	0.28	0.02	0.25	0.56*	0.25	0.49	0.24	0.26	0.55(*)	0.84**
Concentration	ΔE_{max}	0.2 ± 0.29	-0.7 ± 0.37	-0.6 ± 0.32	-0.3 ± 0.45	0.2 ± 0.40	-0.9 ± 0.35	2.10	0.08	0.076	0.47	0.43	0.19	0.02	0.49	0.02	0.13	0.37	0.11	0.15	0.42	0.16	0.2	0.23	0.46
	ΔE_{min}	-2.1 ± 0.4	-2.5 ± 0.4	-2.2 ± 0.4	-2.5 ± 0.5	-2 ± 0.4	-2.9 ± 0.4	0.99	0.04	0.426	0.15	0.07	0.19	0.05	0.35	0.13	0.01	0.27	0.15	0.16	0.17	0.28	0.21	0.15	0.39
Fear	ΔE_{max}	0 ± 0.18	0.2 ± 0.12	0.3 ± 0.41	0.7 ± 0.34	0.2 ± 0.22	1.0 ± 0.3	1.80	0.07	0.109	0.25	0.15	0.37	0.1	0.56(*)	0.04	0.25	0.06	0.44	0.16	0.1	0.25	0.27	0.16	0.41
	ΔE_{min}	-0.2 ± 0.12	0 ± 0.073	-0.3 ± 0.24	-0.2 ± 0.16	-0.2 ± 0.15	0 ± 0.10	0.93	0.04	0.464	0.3	0.1	0.04	0	0.25	0.24	0.25	0.22	0.06	0.07	0.14	0.31	0.07	0.28	0.29

Posthoc comparisons display Cohen's d and p-value: (*)P<0.1, *P<0.05, **P<0.01, ***P<0.001; η_p^2, partial eta-squared; NS, not significant; ΔE_{max}, maximal effect difference from baseline; ΔE_{min}, minimal effect difference from baseline; N=24

Table S4. Mean values and statistics for the acute subjective effects of 2C-B 10, 20 and 30 mg, MDMA, psilocybin and placebo on the 5 Dimensions of Altered States of Consciousness (5D-ASC) Scale

		Placebo	2C-B 10 mg	2C-B 20 mg	2C-B 30 mg	MDMA	Psilocybin	$F_{5,\ 115}$	η_p^2	P=	Pla - 2C-B 10 mg	Pla - 2C-B 20 mg	Pla - 2C-B 30 mg	Pla - MDMA	Pla - Psilo	2C-B 10 mg - 20 mg	2C-B 10 mg - 30 mg	2C-B 10 mg - MDMA	2C-B 10 mg - Psilo	2C-B 20 mg - 30 mg	2C-B 20 mg - MDMA	2C-B 20 mg - Psilo	2C-B 30 mg - MDMA	2C-B 30 mg - Psilo	MDMA - Psilo
		(mean ± SEM)	(mean ± SEM)	(mean ± SEM)	(mean ± SEM)	(mean ± SEM)	(mean ± SEM)																		
5 Dimensions of Altered States of Consciousness (5D-ASC) Scale																									
5D-ASC total Score	% score	1.8 ± 1.1	3.5 ± 0.9	14 ± 2.5	20 ± 2.3	13 ± 1.7	30 ± 2.2	50.14	0.69	<0.001	0.22	1.1***	1.63***	1.58***	2.52***	0.86***	1.43***	1.19***	2.6***	0.5	0.07	1.53***	0.55(*)	1.03***	1.88***
3D-ASC total Score	% score	1.9 ± 1.6	2.5 ± 0.8	15 ± 3.3	21 ± 2.9	15 ± 2.4	33 ± 2.8	42.15	0.65	<0.001	0.07	0.97***	1.53***	1.36***	2.44***	0.79***	1.35***	1.08***	2.46***	0.48	0.03	1.34***	0.47	0.93***	1.79***
Oceanic boundlessness	% score	3.4 ± 3.0	2.7 ± 0.7	19 ± 4.8	29 ± 4.7	28 ± 4.5	38 ± 4.9	21.51	0.48	<0.001	0.05	0.77*	1.31***	1.31***	1.6***	0.68**	1.16***	1.18***	1.56***	0.57	0.41	0.7***	0.04	0.38	0.56
Anxious ego-dissolution	% score	0.2 ± 0.1	0.9 ± 0.4	4.5 ± 1.6	5.2 ± 1.0	2.9 ± 0.6	16 ± 3.2	16.56	0.42	<0.001	0.34	0.57	1	0.89	1.03***	0.46	0.77	0.63	0.95***	0.1	0.25	0.81***	0.43	0.76***	0.88***
Visionary restructuralization	% score	1.5 ± 1.3	4.2 ± 1.4	23 ± 4.5	28 ± 3.7	9.4 ± 2.2	47 ± 3.6	48.44	0.68	<0.001	0.27	1.04***	1.52***	1.03	2.65***	0.85***	1.3***	0.46	2.67***	0.28	0.66**	1.21***	1.11***	1***	2.39***
Auditory alterations	% score	0.0 ± 0.0	0.1 ± 0.0	1.6 ± 0.7	3.3 ± 1.1	0.8 ± 0.3	4.6 ± 1.5	6.59	0.22	<0.001	0.25	0.49	0.6*	0.5	0.63***	0.49	0.6*	0.47	0.62***	0.33	0.23	0.6*	0.47	0.19	0.54**
Reductions of vigilance	% score	3.6 ± 1.3	13 ± 3.3	24 ± 4.5	29 ± 4.4	21 ± 3.7	42 ± 5.1	21.18	0.48	<0.001	0.51	0.93***	1.16***	0.94***	1.48***	0.55(*)	0.9**	0.46	1.12***	0.34	0.29	0.9***	0.49	0.58*	1.03***
3 Dimensions of Altered States of Consciousness revised (3D-ASCr) Scale																									
Positive Effects, PosE	% score	2.4 ± 2.2	2.1 ± 0.7	15 ± 4.3	31 ± 4.5	22 ± 3.7	32 ± 4.6	17.84	0.44	<0.001	0.02	0.7*	1.16***	1.23***	1.47***	0.61*	1.02***	1.14***	1.4***	0.51	0.36	0.63***	0.08	0.34	0.57
Perceptual Effects, PerE	% score	1.9 ± 1.4	5.8 ± 2.1	32 ± 6.7	36 ± 5.1	11 ± 2.6	64 ± 5.1	44.68	0.66	<0.001	0.3	0.98***	1.5***	1.01	2.59***	0.86***	1.27***	0.33	2.43***	0.16	0.68***	1.05***	1.14***	1.08***	2.21***
Disstressing Effects, DisE	% score	0.3 ± 0.2	1.1 ± 0.5	4.9 ± 1.8	5.8 ± 1.2	3.0 ± 0.7	17 ± 3.2	18.87	0.44	<0.001	0.3	0.53	0.95(*)	0.85	1.1***	0.41	0.71	0.58	1***	0.1	0.26	0.89***	0.46	0.81***	0.95***
Experience of unity	% score	3.7 ± 3.7	1.9 ± 0.8	16 ± 5.5	29 ± 5.9	24 ± 5.9	40 ± 6.0	13.97	0.38	<0.001	0.1	0.56	1***	0.83**	1.31***	0.53	0.93***	0.78**	1.34***	0.51	0.27	0.71***	0.15	0.36	0.61*
Spiritual experience	% score	2.8 ± 2.8	0.6 ± 0.4	9.9 ± 4.6	15 ± 4.7	11 ± 3.3	20 ± 5.3	5.68	0.20	<0.001	0.16	0.37	0.62(*)	0.64	0.69**	0.41	0.65*	0.63	0.77***	0.33	0.07	0.35	0.27	0.15	0.4
Blissful state	% score	5.1 ± 4.2	4.0 ± 1.3	24 ± 5.8	38 ± 6.0	51 ± 5.9	39 ± 6.8	18.61	0.45	<0.001	0.05	0.78*	1.2***	1.51***	1.07***	0.68*	1.13***	1.7***	1.11***	0.53	0.91***	0.39	0.35	0.04	0.39
Insightfulness	% score	0.7 ± 0.5	3.0 ± 0.9	21 ± 6.1	25 ± 5.7	24 ± 5.6	33 ± 5.9	10.86	0.32	<0.001	0.43	0.72**	0.89***	0.89***	1.15***	0.62*	0.81**	0.81**	1.04***	0.15	0.12	0.35	0.01	0.26	0.31
Disembodiment	% score	1.3 ± 1.3	2.1 ± 1.2	9.1 ± 3.3	17 ± 3.9	12 ± 3.9	31 ± 6.2	11.94	0.34	<0.001	0.09	0.44	0.86**	0.58	1.05***	0.48	0.81*	0.67	1.04***	0.47	0.14	0.71***	0.18	0.49*	0.77**
Changed meaning	% score	0.6 ± 0.5	1.1 ± 0.5	11 ± 4.0	16 ± 3.7	7.5 ± 2.6	25 ± 3.5	15.57	0.40	<0.001	0.14	0.54*	0.9***	0.56	1.38***	0.48*	0.83***	0.51	1.45***	0.38	0.24	0.63***	0.56	0.47	1.09***
Impaired control and cognition	% score	0.5 ± 0.3	2.1 ± 1.1	6.9 ± 1.8	11 ± 2.2	5.1 ± 1.2	24 ± 3.6	25.77	0.53	<0.001	0.3	0.74(*)	0.94***	0.76	1.37***	0.49	0.69**	0.5	1.19***	0.4	0.26	1.26***	0.51	1***	1.22***
Anxiety	% score	0 ± 0	0 ± 0	3.0 ± 2.3	0.9 ± 0.4	0.8 ± 0.4	10 ± 3.8	5.01	0.18	0.001	0.2	0.26	0.49	0.4	0.56**	0.26	0.46	0.38	0.56**	0.19	0.18	0.38*	0.01	0.51**	0.52**
Complex imagery	% score	3.5 ± 3.5	6.7 ± 2.7	29 ± 6.7	33 ± 6.9	20 ± 5.6	58 ± 6.0	19.74	0.46	<0.001	0.14	0.87**	0.89***	0.79(*)	1.82***	0.67**	0.79**	0.54	1.68***	0.14	0.28	0.84***	0.34	0.62**	1.14***
Elementary imagery	% score	0.6 ± 0.3	4.6 ± 2.4	34 ± 7.9	42 ± 5.7	7.6 ± 2.3	69 ± 5.5	40.38	0.64	<0.001	0.34	0.89***	1.51***	0.63	2.57***	0.85***	1.3***	0.18	2.42***	0.25	0.68***	0.82***	1.25***	0.93***	2.19***
Audio-visual synesthesia	% score	1.5 ± 0.8	6.2 ± 2.5	33 ± 7.3	35 ± 5.8	5.3 ± 1.6	64 ± 7.1	33.71	0.59	<0.001	0.35	0.89***	1.2***	0.57	1.86***	0.83***	0.99***	0.06	1.83***	0.05	0.8***	0.95***	1.12***	0.93***	1.8***

Posthoc comparisons display Cohen's d and p-value: (*)P<0.1, *P<0.05, **P<0.01, ***P<0.001; η_p^2, partial eta-squared; NS, not significant; ΔE_{max}, maximal effect difference from baseline; ΔE_{min}, minimal effect difference from baseline; N=24

Table S5. Mean values and statistics for the acute subjective effects of 2C-B 10, 20 and 30 mg, MDMA, psilocybin and placebo on the Mystical Experience Questionnaire (MEQ) and Psychedelic Experience Scale (PES)

		Placebo (mean ± SEM)	2C-B 10 mg (mean ± SEM)	2C-B 20 mg (mean ± SEM)	2C-B 30 mg (mean ± SEM)	MDMA (mean ± SEM)	Psilocybin (mean ± SEM)	$F_{5,115}$	η_p^2	P=	Pla - 2C-B 10 mg	Pla - 2C-B 20 mg	Pla - 2C-B 30 mg	Pla - MDMA	Pla - Psilo	2C-B 10 mg - 20 mg	2C-B 10 mg - 30 mg	2C-B 10 mg - MDMA	2C-B 10 mg - Psilo	2C-B 20 mg - 30 mg	2C-B 20 mg - MDMA	2C-B 20 mg - Psilo	2C-B 30 mg - MDMA	2C-B 30 mg - Psilo	MDMA - Psilo
Mystical Experiences Questionnaire (MEQ30)																									
Mystical	% score	5.3 ± 4.0	5.3 ± 1.8	20 ± 5.2	25 ± 5.1	23 ± 4.7	30 ± 5.4	11.14	0.33	<0.001	0	0.75*	1***	1.05**	1.08***	0.62*	0.8***	0.83**	0.99***	0.25	0.22	0.43	0.09	0.21	0.42
Positive mood	% score	8.5 ± 3.8	12 ± 2.1	34 ± 4.3	46 ± 4.5	52 ± 4.1	46 ± 5.6	28.46	0.55	<0.001	0.15	1.33***	1.75***	2.15***	1.23***	1.03***	1.44***	2***	1.24***	0.54	0.78**	0.38	0.26	0.01	0.27
Transcendence of time/space	% score	4.7 ± 3.4	6.1 ± 1.7	23 ± 4.4	32 ± 4.7	24 ± 3.8	48 ± 4.5	32.92	0.59	<0.001	0.07	1.11***	1.36***	1.73***	2.05***	0.82***	1.2***	0.91***	1.91***	0.43	0.06	1.38***	0.37	0.72**	1.15***
Ineffability	% score	5.6 ± 4.0	11 ± 3.1	44 ± 5.4	51 ± 5.4	40 ± 5.7	66 ± 4.8	39.76	0.63	<0.001	0.2	1.53***	1.69***	1.31***	2.37***	1.43***	1.63***	1.1***	1.96***	0.22	0.18	0.75**	0.42	0.66*	1***
MEQ30 total score	% score	5.8 ± 3.8	7.3 ± 1.6	26 ± 4.4	33 ± 4.4	31 ± 4.1	41 ± 4.6	25.44	0.52	<0.001	0.07	1.2***	1.5***	1.68***	1.65***	0.88***	1.19***	1.2***	1.5***	0.38	0.33	0.65**	0.12	0.34	0.63
Nadir effects	% score	1.3 ± 0.7	5.0 ± 1.4	9.2 ± 2.7	9.0 ± 1.4	5.1 ± 1.2	19 ± 3.4	10.73	0.32	<0.001	0.44	0.61*	1.29*	0.68	1.02***	0.29	0.49	0.01	0.77***	0.01	0.36	0.6**	0.49	0.55**	0.94***
Aesthetic Experience	% score	5.0 ± 2.1	11 ± 2.2	28 ± 3.8	40 ± 4.5	15 ± 2.1	44 ± 3.9	35.06	0.60	<0.001	0.35	1.37***	1.63***	0.88(*)	1.96***	0.81***	1.26***	0.3	1.79***	0.54*	0.84*	0.76***	1.18***	0.27	1.57***
Mystical Experiences Questionnaire (MEQ43)																									
Internal unity	% score	5.1 ± 3.9	5.0 ± 2.0	20 ± 5.2	25 ± 5.3	21 ± 4.4	29 ± 5.3	10.52	0.31	<0.001	0.01	0.76*	0.91***	1.11**	1.04***	0.62*	0.79***	0.8**	0.93***	0.24	0.12	0.37	0.14	0.18	0.38
External unity	% score	3.6 ± 2.8	3.8 ± 1.3	15 ± 4.0	24 ± 4.9	18 ± 4.3	30 ± 4.9	14.22	0.38	<0.001	0.01	0.82*	1.09***	1.14**	1.28***	0.64*	0.9***	0.66**	1.08***	0.47	0.19	0.69**	0.28	0.23	0.6*
Sacredness	% score	5.5 ± 3.8	5.0 ± 1.5	21 ± 5.4	30 ± 5.2	26 ± 4.5	31 ± 6.1	11.81	0.34	<0.001	0.02	0.73*	1.17***	1.26***	0.93***	0.66*	0.98***	1***	0.93***	0.36	0.27	0.35	0.18	0.06	0.24
Noetic quality	% score	5.4 ± 4.2	8.8 ± 2.8	22 ± 5.6	30 ± 5.5	25 ± 5.1	36 ± 5.2	12.19	0.35	<0.001	0.13	0.73*	1.04***	1.02**	1.39***	0.51(*)	0.75***	0.66*	1.09***	0.31	0.16	0.51(*)	0.21	0.26	0.66
Deeply felt positive mood	% score	7.5 ± 3.5	12 ± 2.4	34 ± 4.3	43 ± 5.0	55 ± 5.1	45 ± 5.7	27.04	0.54	<0.001	0.21	1.4***	1.58***	1.85***	1.27***	0.98***	1.26***	1.76***	1.25***	0.4	0.73**	0.36	0.44	0.09	0.47
Transcendence of time/space	% score	4.6 ± 3.5	5.1 ± 1.5	22 ± 4.6	30 ± 4.8	23 ± 4.1	45 ± 4.7	27.70	0.55	<0.001	0.03	1.02***	1.27***	1.43***	1.89***	0.76***	1.11***	0.85***	1.83***	0.36	0.03	1.21***	0.34	0.65**	1.16***
Ineffability	% score	4.7 ± 3.3	7.8 ± 2.4	34 ± 4.8	41 ± 5.0	30 ± 4.9	53 ± 4.8	33.37	0.59	<0.001	0.15	1.57***	1.65***	1.23***	2.06***	1.19***	1.46***	0.96***	1.75***	0.29	0.25	0.73**	0.43	0.48	1.03***
Psychedelic Experience Questionnaire/Scale (PES)																									
Paradoxicality	% score	3.3 ± 2.7	2.3 ± 1.2	17 ± 4.4	23 ± 5.2	13 ± 4.1	31 ± 4.6	16.37	0.42	<0.001	0.07	0.93*	0.96***	0.76	1.44***	0.65**	0.82***	0.5(*)	1.26***	0.32	0.41	0.74**	0.48(*)	0.4	1.07***
Connectedness	% score	8.5 ± 4.1	16 ± 3.1	34 ± 4.9	44 ± 5.1	41 ± 5.4	47 ± 5.0	19.42	0.46	<0.001	0.25	1.01***	1.31***	1.24***	1.38***	0.78**	1.1***	0.96***	1.3***	0.39	0.33	0.57(*)	0.12	0.14	0.36
Closeness With Guide	% score	5.8 ± 3.3	5.8 ± 2.2	23 ± 5.3	28 ± 6.4	32 ± 6.8	34 ± 6.0	9.75	0.30	<0.001	0	0.96*	0.94**	0.85***	0.89***	0.64*	0.69**	0.89***	0.91***	0.31	0.36	0.37	0.12	0.17	0.08
Visual Experience	% score	2.5 ± 1.3	6.9 ± 1.7	24 ± 4.0	35 ± 4.9	4.4 ± 1.3	42 ± 3.8	36.41	0.61	<0.001	0.38	1.06***	1.37***	0.28	2.07***	0.8***	1.14***	0.25	1.99***	0.46(*)	0.98***	0.88***	1.33***	0.34	2.03***
Distressing Experience	% score	1.8 ± 1.1	6.7 ± 1.8	8.2 ± 3.0	8.8 ± 2.5	3.7 ± 1.6	19 ± 5.4	5.05	0.18	<0.001	0.45	0.43	0.71	0.24	0.65***	0.09	0.16	0.22	0.44*	0.04	0.33	0.43(*)	0.36	0.36(*)	0.7**
Physical distress	% score	2.9 ± 1.8	12 ± 3.0	20 ± 4.5	21 ± 3.4	14 ± 3.4	26 ± 5.3	6.29	0.21	<0.001	0.52	0.76**	0.96**	0.72	0.97***	0.36	0.48	0.08	0.47*	0.05	0.23	0.21	0.32	0.21	0.5(*)
Cognition	% score	2.0 ± 1.3	7.0 ± 2.1	16 ± 2.5	22 ± 4.0	15 ± 2.6	32 ± 3.6	20.68	0.47	<0.001	0.38	1.24***	1.12***	0.99**	1.69***	0.61(*)	0.73***	0.59	1.32***	0.35	0.04	0.99***	0.34	0.64(*)	1.06***
Affect	% score	3.5 ± 2.0	6.3 ± 1.6	23 ± 3.4	27 ± 4.3	34 ± 4.1	29 ± 3.9	22.06	0.49	<0.001	0.2	1.56***	1.31***	1.5***	1.29***	0.95***	1.03***	1.52***	1.15***	0.26	0.55*	0.3	0.35	0.11	0.29

Posthoc comparisons display Cohen's d and p-value: (*)P<0.1, *P<0.05, **P<0.01, ***P<0.001; η_p^2, partial eta-squared; NS, not significant; ΔE_{max}, maximal effect difference from baseline; ΔE_{min}, minimal effect difference from baseline; N=24

Table S6. Acute adverse drug effects after administration of 2C-B 10, 20 and 30 mg, MDMA, psilocybin, and placebo.

	Placebo		2C-B 10 mg		2C-B 20 mg		2C-B 30 mg		Psilocybin		MDMA	
	0h	0-9h	0h	0-9h	0h	0-9h	0h	0-9h	0h	0-9h	0h	0-9h
Fatigue	9	13	11	20	6	18	7	19	8	19	7	20
Headache	2	6	2	15	3	13	1	13	1	11	1	14
Feeling dull	2	5	0	14	2	10	2	11	1	12	2	13
Lack of energy	1	3	1	8	0	7	0	10	0	13	0	11
Lack of concentration	0	2	0	5	0	9	0	9	0	15	0	7
Decreased appetite	1	1	1	3	0	8	2	9	1	12	1	13
Nausea	0	1	0	6	0	11	0	13	0	8	0	5
Dry mouth	0	1	0	5	0	7	0	11	0	9	0	8
Increased need to sleep	0	3	0	6	0	7	0	5	0	8	0	8
Impaired balance	0	0	0	4	0	5	0	8	0	9	0	3
Bruxism	0	0	0	4	0	3	0	7	0	4	0	11
Feeling exhausted	0	2	0	4	0	4	0	6	0	9	0	6
Feeling of weakness	0	1	0	2	1	6	1	5	0	9	0	6
Job-related or personal worries	4	5	3	5	0	4	1	5	3	9	3	5
Feeling restless	0	1	0	2	0	5	0	9	1	8	0	3
Restless legs	0	0	0	1	0	7	0	6	0	7	0	6
Tremor	0	1	0	0	0	7	1	7	0	5	0	5
Cold feet	0	2	1	4	0	4	2	5	1	5	3	6
Inner tension	1	1	2	1	2	5	2	4	3	10	4	4
Obsessive rumination	0	1	0	2	0	4	0	6	1	8	0	3
Freezing	0	2	0	3	0	3	0	6	0	8	1	3
Hypersensitivity to cold	0	2	0	3	0	4	0	5	0	6	0	4
Tendency to cry	0	0	0	1	0	5	0	4	0	9	0	2
Dizziness	0	0	0	2	0	3	0	6	0	6	0	1
Forgetfulness	0	1	1	0	0	4	0	6	0	5	0	3
Neck pain	1	2	1	4	0	2	1	5	0	3	1	4
Hypersensitivity to certain odors	0	1	1	2	0	6	0	2	0	6	0	1
Perspiration	0	0	0	2	0	2	0	2	0	4	0	7
Palpitations	0	1	0	1	0	4	0	1	0	5	0	6
Throat tightness	2	0	0	1	0	3	0	6	0	4	0	2
Anxiety	0	0	0	0	0	4	0	4	0	8	0	0
Erotic thoughts	0	1	0	2	0	3	0	3	0	3	0	4
Micturition urgency	0	1	0	2	0	2	0	2	0	4	0	5
Abdominal pain	0	0	1	1	0	4	1	3	1	6	0	0
Hot flashes / flushing	0	0	0	1	1	1	1	3	0	5	0	4
Negative thoughts	0	0	0	2	0	1	0	4	0	6	0	0
Hypersensitivity to warm	0	0	0	1	0	4	0	3	0	3	0	1
Heavy legs	0	1	0	0	0	2	0	2	0	2	0	3
Increased appetite	1	1	0	2	0	5	0	2	0	0	0	0
Not feeling at ease	0	0	0	0	0	1	0	3	0	3	0	0
Dyspepsia	0	0	0	0	0	2	0	2	0	1	0	2
Feeling of pressure or abdominal fullness	0	1	0	1	0	2	0	0	0	3	0	0
Constipation	0	0	0	1	0	0	0	1	0	2	0	1
Dyspnoea	0	0	0	0	0	1	0	2	0	1	0	0
Back pain	0	0	0	1	0	0	0	0	1	2	0	1
Insomnia	1	0	1	0	0	0	0	1	1	1	1	1
Vomiting	0	0	0	1	0	0	0	1	0	0	0	1
Cough	0	1	0	1	1	1	0	0	0	1	0	0
Sore/scratchy throat	2	0	0	1	0	2	0	0	1	0	0	0
Irritability	0	0	0	1	0	2	0	0	0	0	0	0
Numbness in hands or feet	0	1	0	1	0	2	0	0	0	0	0	0
Joint and limb pain	1	1	0	0	0	0	0	1	0	1	0	0
Decreased libido	0	0	0	0	0	0	0	1	0	1	0	0
Feeling suffocated	0	0	0	0	0	1	0	1	0	0	0	0
Dysphagia	1	1	0	0	0	0	0	1	0	0	0	1
Hiccups	0	0	0	0	0	0	0	1	0	0	0	1
Chest pain	0	0	0	2	0	0	0	0	0	0	0	0
Diarrhea	0	0	0	0	0	0	0	0	0	0	0	2
Intermittent shortness of breath	0	0	0	0	0	1	0	0	0	0	0	0
Fear of death	0	0	0	0	0	1	0	0	0	0	0	0
Decrease in body weight	1	0	0	0	2	0	0	0	0	0	0	1

Data indicate number of subjects reporting an effect among a total of 24 subjects

Table S7. Assessment of blinding

	2C-B 10 mg (N = 24)			2C-B 20 mg (N = 24)			2C-B 30 mg (N = 24)		
	during session	after session	after study	during session	after session	after study	during session	after session	after study
correctly identified	4 (17%)	13 (54%)	17 (71%)	3 (13%)	14 (58%)	16 (67%)	3 (13%)	10 (42%)	9 (38%)
mistaken for 2C-B 10 mg				4 (17%)	2 (8%)	1 (4%)	5 (21%)	3 (13%)	1 (4%)
mistaken for 2C-B 20 mg	7 (29%)	3 (13%)	1 (4%)				5 (21%)	2 (8%)	2 (8%)
mistaken for 2C-B 30 mg	2 (8%)	0 (0%)	2 (8%)	3 (13%)	1 (4%)	4 (%)			
mistaken for MDMA	3 (13%)	2 (8%)	1 (4%)	5 (21%)	3 (13%)	1 (4%)	3 (13%)	0 (0%)	2 (8%)
mistaken for Psilocybin	3 (13%)	0 (0%)	0 (0%)	5 (21%)	4 (17%)	1 (4%)	5 (21%)	9 (38%)	9 (38%)
mistaken for Placebo	5 (21%)	6 (%)	3 (13%)	4 (17%)	0 (0%)	1 (4%)	3 (13%)	0 (0%)	1 (4%)
	MDMA (N = 24)			Psilocybin (N = 24)			Placebo (N = 24)		
	during session	after session	after study	during session	after session	after study	during session	after session	after study
correctly identified	3 (13%)	19 (79%)	19 (79%)	4 (17%)	11 (46%)	13 (54%)	4 (17%)	16 (67%)	19 (79%)
mistaken for 2C-B 10 mg	4 (17%)	1 (4%)	3 (13%)	6 (25%)	0 (0%)	0 (0%)	5 (21%)	7 (29%)	2 (8%)
mistaken for 2C-B 20 mg	3 (13%)	4 (17%)	1 (4%)	3 (13%)	3 (13%)	3 (13%)	4 (17%)	1 (4%)	1 (4%)
mistaken for 2C-B 30 mg	5 (21%)	0 (0%)	1 (4%)	3 (13%)	10 (42%)	7 (29%)	2 (8%)	0 (0%)	1 (4%)
mistaken for MDMA				5 (21%)	0 (0%)	1 (4%)	5 (21%)	0 (0%)	0 (0%)
mistaken for Psilocybin	4 (17%)	0 (0%)	0 (0%)				4 (17%)	0 (0%)	1 (4%)
mistaken for Placebo	5 (21%)	0 (0%)	0 (0%)	3 (13%)	0 (0%)	0 (0%)			

Section 3 Consort Flow-Chart

Consort flow chart

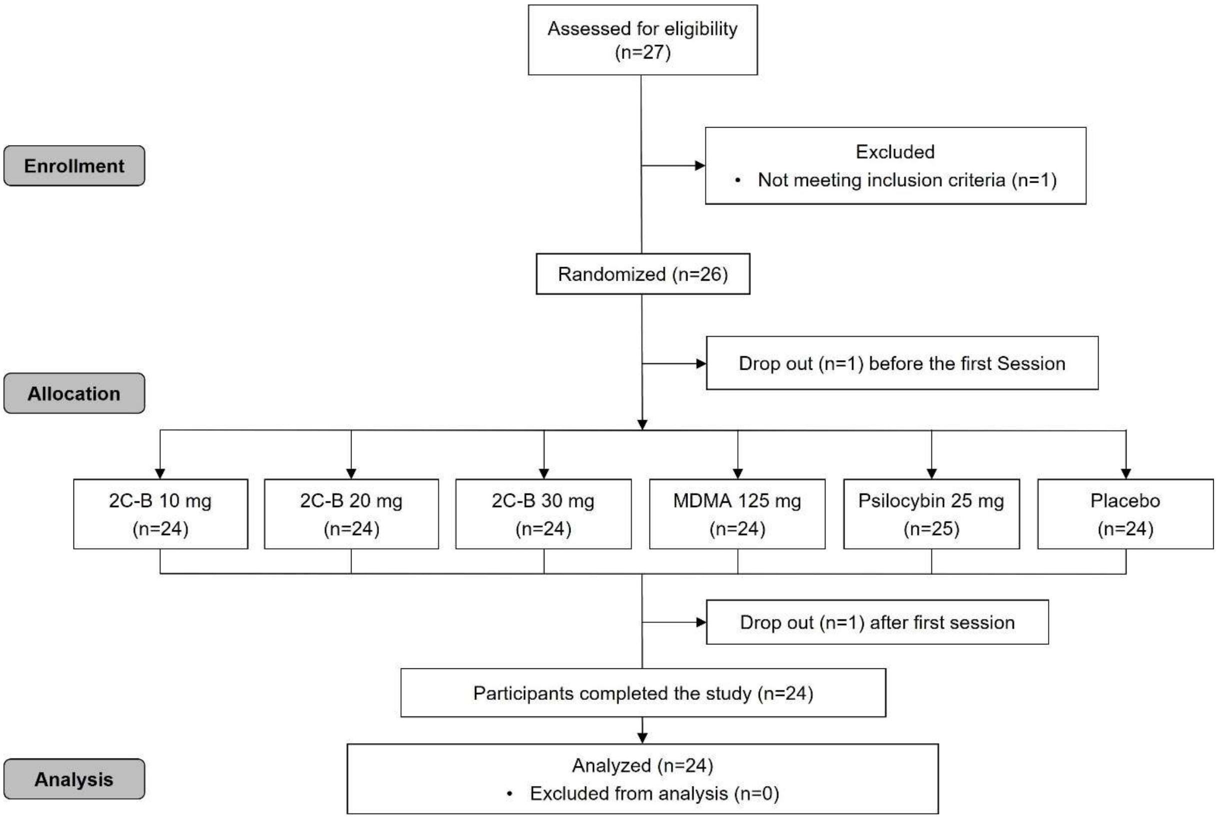

Section 4 References

1 Holze F, Vizeli P, Muller F, Ley L, Duerig R, Varghese N, et al. Distinct acute effects of LSD, MDMA, and D-amphetamine in healthy subjects. Neuropsychopharmacology. 2020;45(3):462-71.

2 Schmid Y, Enzler F, Gasser P, Grouzmann E, Preller KH, Vollenweider FX, et al. Acute effects of lysergic acid diethylamide in healthy subjects. Biol Psychiatry. 2015;78(8):544-53.

3 Hysek CM, Schmid Y, Simmler LD, Domes G, Heinrichs M, Eisenegger C, et al. MDMA enhances emotional empathy and prosocial behavior. Soc Cogn Affect Neurosci. 2014;9:1645-52.

4 Ley L, Holze F, Arikci D, Becker AM, Straumann I, Klaiber A, et al. Comparative acute effects of mescaline, lysergic acid diethylamide, and psilocybin in a randomized, double-blind, placebo-controlled cross-over study in healthy participants. Neuropsychopharmacology. 2023;48(11):1659-67.

5 Straumann I, Ley L, Holze F, Becker AM, Klaiber A, Wey K, et al. Acute effects of MDMA and LSD co-administration in a double-blind placebo-controlled study in healthy participants. Neuropsychopharmacology. 2023;48(13):1840-48.

6 Holze F, Ley L, Muller F, Becker AM, Straumann I, Vizeli P, et al. Direct comparison of the acute effects of lysergic acid diethylamide and psilocybin in a double-blind placebo-controlled study in healthy subjects. Neuropsychopharmacology. 2022;47(6):1180-87.

7 Janke W, Debus G. Die Eigenschaftswörterliste. Hogrefe: Göttingen.; 1978.

8 Dittrich A. The standardized psychometric assessment of altered states of consciousness (ASCs) in humans. Pharmacopsychiatry. 1998;31 (Suppl 2):80-4.

9 Studerus E, Gamma A, Vollenweider FX. Psychometric evaluation of the altered states of consciousness rating scale (OAV). PLoS One. 2010;5(8):e12412.

10 Stocker K, Hartmann M, Schmid Y, Vogt SB, Becker A, Ley L, et al. The 3D-ASCr scale: a revalidation of the core dimensions of the altered states of consciousness rating scale 5D(11)-ASC for psychedelic research. J Psychopharmacol. 2025;in press; doi: 10.1177/02698811251397328.

11 Liechti ME, Dolder PC, Schmid Y. Alterations in conciousness and mystical-type experiences after acute LSD in humans. Psychopharmacology. 2017;234:1499-510.

12 Carhart-Harris RL, Kaelen M, Bolstridge M, Williams TM, Williams LT, Underwood R, et al. The paradoxical psychological effects of lysergic acid diethylamide (LSD). Psychol Med. 2016;46:1379-90.

13 Dolder PC, Schmid Y, Mueller F, Borgwardt S, Liechti ME. LSD acutely impairs fear recognition and enhances emotional empathy and sociality. Neuropsychopharmacology. 2016;41:2638-46.

14 Bershad AK, Schepers ST, Bremmer MP, Lee R, de Wit H. Acute subjective and behavioral effects of microdoses of lysergic acid diethylamide in healthy human volunteers. Biol Psychiatry. 2019;86(10):792-800.

15 Preller KH, Herdener M, Pokorny T, Planzer A, Kraehenmann R, Stämpfli P, et al. The fabric of meaning and subjective effects in LSD-induced states depend on serotonin 2A receptor activation Curr Biol. 2017;27:451-57.

16 Schmid Y, Gasser P, Oehen P, Liechti ME. Acute subjective effects in LSD- and MDMA-assisted psychotherapy. J Psychopharmacol. 2021;35(4):362-74.

17 Roseman L, Nutt DJ, Carhart-Harris RL. Quality of acute psychedelic experience predicts therapeutic efficacy of psilocybin for treatment-resistant depression. Front Pharmacol. 2017;8:974.

18 Griffiths RR, Johnson MW, Carducci MA, Umbricht A, Richards WA, Richards BD, et al. Psilocybin produces substantial and sustained decreases in depression and anxiety in patients with life-threatening cancer: a randomized double-blind trial. J Psychopharmacol. 2016;30(12):1181-97.

19 Holze F, Gasser P, Muller F, Dolder PC, Liechti ME. Lysergic acid diethylamide-assisted therapy in patients with anxiety with and without a life-threatening illness: a randomized, double-blind, placebo-controlled phase II study. Biol Psychiatry. 2023;93(3):215-23.

20 Stocker K, Hartmann M, Ley L, Becker AM, Holze F, Liechti ME. The revival of the psychedelic experience scale: revealing its extended-mystical, visual, and distressing experiential spectrum with LSD and psilocybin studies. J Psychopharmacol. 2024;38(1):80-100.

21 Griffiths RR, Richards WA, McCann U, Jesse R. Psilocybin can occasion mystical-type experiences having substantial and sustained personal meaning and spiritual significance. Psychopharmacology. 2006;187(3):268-83; discussion 84-92.

22 Barrett FS, Johnson MW, Griffiths RR. Validation of the revised Mystical Experience Questionnaire in experimental sessions with psilocybin. J Psychopharmacol. 2015;29(11):1182-90.

23 MacLean KA, Johnson MW, Griffiths RR. Mystical experiences occasioned by the hallucinogen psilocybin lead to increases in the personality domain of openness. J Psychopharmacol. 2011;25(11):1453-61.

24 Griffiths RR, Johnson MW, Richards WA, Richards BD, McCann U, Jesse R. Psilocybin occasioned mystical-type experiences: immediate and persisting dose-related effects. Psychopharmacology. 2011;218(4):649-65.

25 Griffiths R, Richards W, Johnson M, McCann U, Jesse R. Mystical-type experiences occasioned by psilocybin mediate the attribution of personal meaning and spiritual significance 14 months later. J Psychopharmacol. 2008;22(6):621-32.

26 Garcia-Romeu A, Griffiths RR, Johnson MW. Psilocybin-occasioned mystical experiences in the treatment of tobacco addiction. Curr Drug Abuse Rev. 2014;7(3):157-64.

27 Garcia-Romeu A, Davis AK, Erowid F, Erowid E, Griffiths RR, Johnson MW. Cessation and reduction in alcohol consumption and misuse after psychedelic use. J Psychopharmacol. 2019;33(9):1088-101.

28 Griffiths RR, Johnson MW, Richards WA, Richards BD, Jesse R, MacLean KA, et al. Psilocybin-occasioned mystical-type experience in combination with meditation and other spiritual practices produces enduring positive changes in psychological functioning and in trait measures of prosocial attitudes and behaviors. J Psychopharmacol. 2018;32:49-69.

29 Ross S, Bossis A, Guss J, Agin-Liebes G, Malone T, Cohen B, et al. Rapid and sustained symptom reduction following psilocybin treatment for anxiety and depression in patients with life-threatening cancer: a randomized controlled trial. J Psychopharmacol. 2016;30(12):1165-80.

30 Becker AM, Klaiber A, Holze F, Istampoulouoglou I, Duthaler U, Varghese N, et al. Ketanserin reverses the acute response to LSD in a randomized, double-blind, placebo-controlled, crossover study in healthy participants. Int J Neuropsychopharmacol. 2023;26(2):97-106.

31 Holze F, Vizeli P, Ley L, Muller F, Dolder P, Stocker M, et al. Acute dose-dependent effects of lysergic acid diethylamide in a double-blind placebo-controlled study in healthy subjects. Neuropsychopharmacology. 2021;46(3):537-44.

32 Dziobek I, Rogers K, Fleck S, Bahnemann M, Heekeren HR, Wolf OT, et al. Dissociation of cognitive and emotional empathy in adults with Asperger syndrome using the Multifaceted Empathy Test (MET). J Autism Dev Disord. 2008;38(3):464-73.

33 Schmid Y, Hysek CM, Simmler LD, Crockett MJ, Quednow BB, Liechti ME. Differential effects of MDMA and methylphenidate on social cognition. J Psychopharmacol. 2014;28:847-56.

34 Holze F, Avedisian I, Varghese N, Eckert A, Liechti ME. Role of the 5-HT2A receptor in acute effects of LSD on empathy and circulating oxytocin. Front Pharmacol. 2021;12:711255.

35 Pokorny T, Preller KH, Kometer M, Dziobek I, Vollenweider FX. Effect of psilocybin on empathy and moral decision-making. Int J Neuropsychopharmacol. 2017;20:747-57.

36 Bedi G, Hyman D, de Wit H. Is ecstasy an "empathogen"? Effects of ±3,4-methylenedioxymethamphetamine on prosocial feelings and identification of emotional states in others. Biol Psychiatry. 2010;68:1134-40.

37 Dolder PC, Muller F, Schmid Y, Borgwardt SJ, Liechti ME. Direct comparison of the acute subjective, emotional, autonomic, and endocrine effects of MDMA, methylphenidate, and modafinil in healthy subjects. Psychopharmacology (Berl). 2018;235(2):467-79.

38 Dolder PC, Strajhar P, Vizeli P, Odermatt A, Liechti ME. Acute effects of lisdexamfetamine and D-amphetamine on social cognition and cognitive performance in a placebo-controlled study in healthy subjects. Psychopharmacology (Berl). 2018;235(5):1389-402.
